# Comparative meta-analysis of robot- and video-assisted surgery for thymoma: efficacy, learning curve, and economic burden in 7347 patients

**DOI:** 10.1186/s12957-025-04132-2

**Published:** 2025-11-29

**Authors:** Guohang Shen, Yupei Dai, Yaxu Xin, Kaiyong Wang, Haobo Zhang, Yang Chen, Linyun Wang, Zongxin Li, Feng Li, Fengli Gao

**Affiliations:** 1 Department of Clinical Medicine, Ningxia Medical University, Yinchuan, 750004 Ningxia Hui Autonomous Region PR China; 2 General Hospital of Ningxia Medical University, 804 Shengli South Street, Yinchuan, Ningxia Hui Autonomous Region 750004 PR China

**Keywords:** Robot-assisted surgery, Thymoma, Video-assisted surgery, Thymectomy, Clinical outcomes

## Abstract

**Objective:**

This meta-analysis systematically evaluates the perioperative outcomes, learning curve, and hospitalization costs of robot-assisted thoracoscopic surgery (RATs) compared to video-assisted thoracoscopic surgery (VATs) for thymoma resection.

**Methods:**

A comprehensive literature search was conducted in PubMed, Embase, Web of Science, and the Cochrane Library, with the final update in June 2025, following PRISMA guidelines. Constant-effects model or random-effects model was used based on heterogeneity, and subgroup analyses were performed accordingly.

**Results:**

A total of 30 studies involving 7,347 patients (RATs: 3,122; VATs: 4,225) were included, and 17 outcome indicators were analyzed. Compared with VATs, RATs showed lower conversion rates, reduced blood loss, shorter chest tube duration, and fewer complications—including pulmonary infections—while operative time and hospital stay differences were modest. Analysis of the learning curve in 153 RATs patients showed operative times significantly decreased after 20 procedures. Total hospitalization costs were higher for RATs, there were no significant differences in 30-day or 90-day mortality between groups.

**Conclusion:**

RATs showed favorable perioperative outcomes, including faster recovery and fewer complications, indicating potential clinical advantages in thymoma surgery. Although associated with higher hospitalization costs, further large-scale prospective studies are needed to confirm its broader applicability.

**Supplementary Information:**

The online version contains supplementary material available at 10.1186/s12957-025-04132-2.

## Introduction

Thymectomy remains the gold standard for thymic diseases, especially primary tumors, thymic cysts, suspected anterior mediastinal masses, and myasthenia gravis (MG) [1]. The principal goal in the management of thymic pathologies, particularly thymoma and thymic cysts, is complete surgical excision to alleviate mass effect and achieve optimal disease control. For localized or early-stage thymoma, surgical resection remains the standard and most effective treatment, which can be performed through thoracoscopic, robot-assisted, or conventional open approaches [2]. This procedure not only ensures complete tumor removal but also prolongs survival, particularly in the absence of distant metastasis [3]. For patients with localized thymoma, surgical resection remains the treatment of choice, and minimally invasive techniques, such as thoracoscopic or robot-assisted surgery, can significantly reduce postoperative recovery time and the risk of complications [4]. However, no consensus has been reached on the optimal surgical technique, and the choice often depends on the surgeon’s experience and judgment [5].

With the increasing adoption of RATs, RATs has become an increasingly utilized technique for thymectomy. Compared with conventional VATs, the da Vinci Surgical System offers superior maneuverability through articulated instruments, enabling precise dissection in confined anatomical areas [6]. The robotic instrument’s wrist mechanism enhances precision, allowing for greater flexibility during surgery [7, 8]. Furthermore, the system’s capability to filter out physiological tremors enhances surgical precision and reduces intraoperative risk, thereby improving overall procedural quality [9, 10].

Although a few meta-analyses have compared the perioperative outcomes of different thymectomy techniques for thymoma—including operative time, hospital stay, blood loss, and the need for adjuvant therapy—they were limited by small sample sizes and narrow literature coverage [11, 12]. Moreover, these studies mainly focused on traditional clinical endpoints, overlooking key aspects such as R0 resection rate, hospitalization costs, surgeons’ learning curves, and outcomes of the full lateral approach. The present meta-analysis addresses these gaps by comparing perioperative outcomes, learning curves, and economic factors between RATs and VATs, emphasizing clinical efficacy, complications, and postoperative recovery.

## Methods

### Literature and database search strategy

We conducted this systematic review and meta-analysis in accordance with PRISMA guidelines and was preregistered on PROSPERO, https://www.crd.york.ac.uk/PROSPERO/ (registration no. CRD420251118017) [13]. Up to June 2025, relevant literature was retrieved from PubMed (biomedical database), the Cochrane Library, Embase and Web of Science. Target studies were identified using controlled vocabulary and keywords including “thymoma,” “robot-assisted,” and “video-assisted.” For example, the PubMed search string was: ((((thymoma) OR (Thymoma)) OR (mediastinal tumor)) OR (mediastinal)) AND ((((((((da Vinci) OR (Robot)) OR (robot)) OR (robotic)) OR (robot-assisted)) OR (robotic-assisted)) OR (robotic-assisted thoracic surgery)) AND (((((((vat) OR (VATs)) OR (VAT)) OR (Thoracoscope)) OR (video assisted thoracic surgery)) OR (video)) OR (thoracoscopic))).Only English-language publications were included in the analysis. Citation lists from the relevant clinical studies and meta-analyses were examined manually to identify additional applicable reports. Two reviewers independently screened and assessed the initially retrieved records. To avoid duplication of data from the same cohort, when multiple reports originated from a single study, only the most comprehensive or most recent report was included.

### Eligibility criteria and data extraction

Eligibility criteria were established according to the PICOS framework (Population, Intervention, Comparison, Outcomes, Study design) to ensure methodological rigor and result validity [14]. Two reviewers independently screened full texts and extracted data; any discrepancies were resolved by consensus with a third reviewer. Specifically: P (Population): patients undergoing thymoma resection. I (Intervention): robot-assisted thymectomy. C (Comparison): video-assisted thymectomy. O (Outcomes): perioperative clinical outcomes, postoperative complications, and costs. S (Study design): randomized trials alongside cohort-based investigations. Using these criteria, we selected high-quality, relevant studies. Extracted information included study characteristics: publication year; surgical category; sample size; patient demographics (age, sex, body mass index (BMI); tumor size; world health organization (WHO)histological classification; surgical approach; MG status). Perioperative outcomes: conversion rate to open surgery; operative time; intraoperative blood loss; drainage volume and duration; R0 resection rate; postoperative length of stay; complication rates (including pulmonary infection); total hospitalization costs; learning-curve metrics; and 30- and 90-day mortality. Studies were excluded if they were duplicates, non-comparative designs (case reports, reviews, conference abstracts, meta-analyses, single-arm studies), not published in English, or lacked sufficient or high-quality data.

### Quality assessment, publication bias, and statistical analysis

Study quality was evaluated using the Newcastle-Ottawa Scale (NOS), which assesses selection, comparability, and exposure, with a star-based system (maximum 9 stars); studies with >6 stars were deemed of acceptable quality and those with 8–9 stars high quality. Quantitative data required for the meta-analysis—such as means, standard deviations (SDs), mean differences (MDs), and odds ratios (ORs) were either extracted directly or derived via calculation, MD stands for mean difference, which represents the difference in the means of two groups. When applicable, units are specified (e.g., minutes, milliliters), and for ratios, MD indicates the comparative effect size without units [15]. When only medians and interquartile ranges were reported, they were converted to means and SDs using the Hozo method [16]. Study-specific ORs and their 95% confidence intervals (CIs) were calculated. Heterogeneity was assessed with the I² statistic, with < 25%, 25–50%, and >50% representing low, moderate, and high heterogeneity, respectively. A random-effects model was used if I² >50%; otherwise, a fixed-effect model was applied, with the fixed-effect results also examined in sensitivity analyses [17]. Dichotomous outcomes were summarized as ORs with 95% CI, and continuous outcomes as MDs with 95% CI [18, 19]. Statistical processing was carried out using the Review Manager program (version 5.4; Cochrane Collaboration, Oxford, UK). Funnel plots were visually examined to assess possible publication bias, and subgroup analyses limited to patients treated with the lateral approach—A fully lateral approach refers to a surgical positioning where the patient is placed in a lateral decubitus position, allowing the surgeon to access the thymus via the chest wall without the need for a sternotomy. This differs from other approaches, such as subxiphoid or median sternotomy, which may involve a different orientation of the patient or entry point [20]. Two-sided P values < 0.05 were considered statistically significant.

## Results

### Study selection and characteristics

**Fig. 1:**
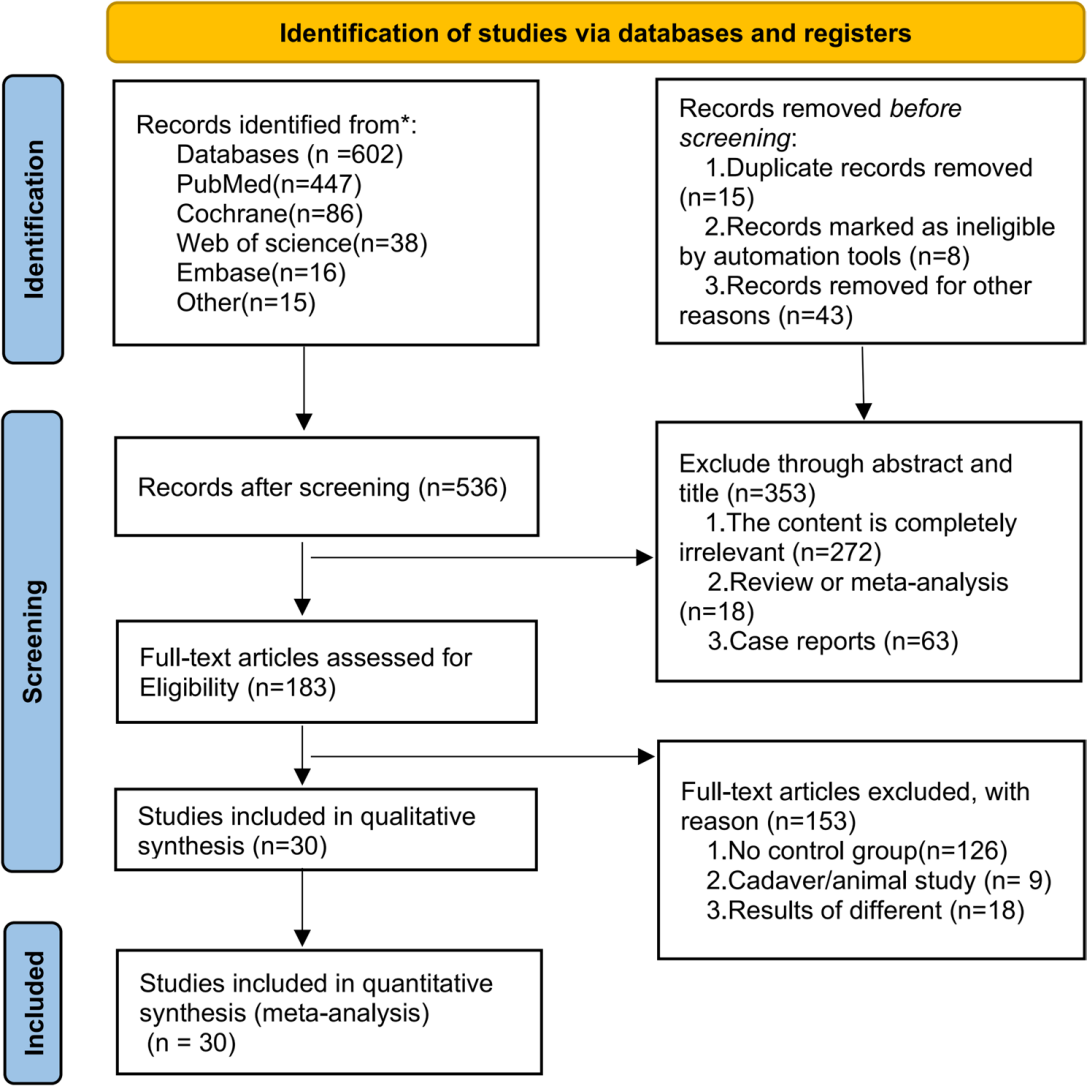
Research options flow chart

A total of 602 articles were identified through database and manual searches. After careful full-text screening (with reasons for exclusions outlined in Fig. 1). A total of 30 retrospective studies, including 17 outcomes and 7,347 patients, were analyzed. Of these, 3,122 patients underwent RATs (experimental group) and 4,225 received VATs (control group). The studies, spanning from 2013 to 2025, have over 77% published in the last five years [4, 21–49]. Table 3 provides a summary of the patients’ baseline characteristics, and detailed outcome data from 30 studies are presented in Table 4. It revealed that the median age ranged from 50 to 60 years, with males accounting for 50.36% and females 49.63%. The median tumor size averaged 4.5 cm, the prevalence of MG was approximately 20%, and 93.77% of cases underwent a lateral surgical approach. The risk of bias for the included studies is shown in Table 2, evaluated using the Newcastle-Ottawa Scale (NOS). Most studies had a low risk of bias, with only a few having methodological limitations. The detailed risk of bias for each study is presented in Table 1, highlighting the potential impact on the findings.

### Conversion to open surgery

**Fig. 2:**
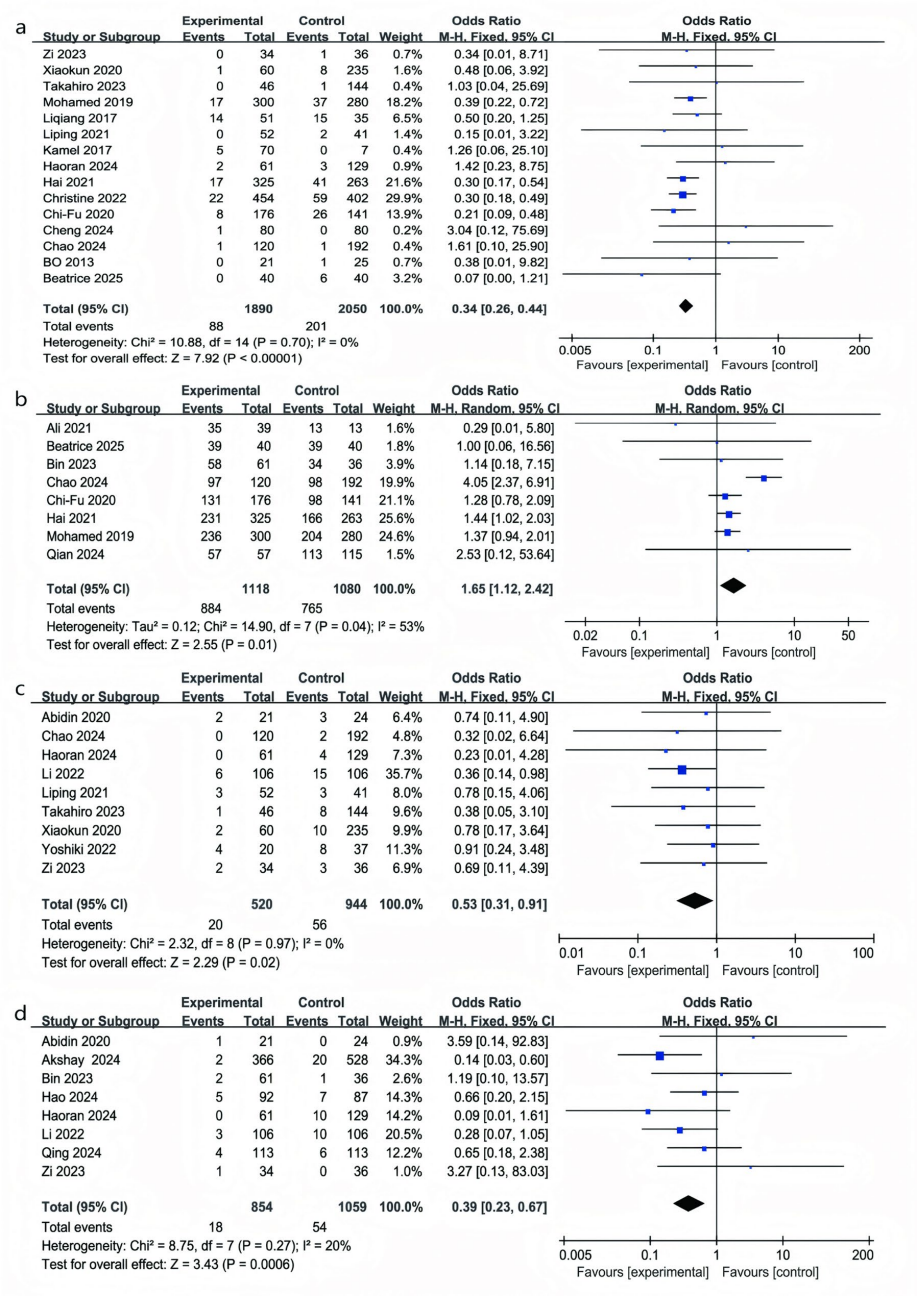
Forest plots comparing conversion rates, R0 resection rates, overall complications, and pulmonary infection rates between RATs and VATs (panels **a**-**d**)

A meta-analysis of 15 studies involving 3,940 patients (RATs: 1,890; VATs: 2,050) revealed a significantly lower conversion rate to open surgery in the RATs group. Specifically, patients undergoing RATs had about a two-thirds lower risk of conversion compared with those receiving VATs (OR 0.34, 95% CI 0.26–0.44; *P* < 0.05; I² = 0%) (Fig. 2a) [22, 27–32, 34, 35, 37–39, 43, 45, 49].

### R0 resection

In eight studies including 2,198 patients (RATs: 1,118; VATs: 1,080), the rate of complete (R0) resection was significantly higher in the RATs group. Patients treated with RATs had about a 65% greater chance of achieving R0 resection compared with VATs (OR = 1.65; 95% CI: 1.12–2.42; *P* = 0.01). Between-study variability was moderate (I² = 53%; *P* = 0.04), but the overall trend consistently favored RATs (Fig. 2b) [22, 24, 26, 28, 30, 32, 39, 41].

### Overall complications

Incorporating data from 9 studies with 1,464 patients (RATs: 520; VATs: 944), overall complications, including arrhythmias, wound infections, phrenic nerve injury, intercostal neuralgia, and pneumothorax, were assessed. The results showed that the likelihood of developing postoperative complications was reduced by nearly half in RATs compared with VATs (OR 0.53, 95% CI 0.31–0.91; *P* = 0.02; I² = 0%) (Fig. 2c) [23, 28, 34, 36, 37, 43, 45, 47, 49].

### Pulmonary infection

We separately analyzed pulmonary infections, as they are a common and clinically significant complication after thymectomy. Pulmonary infections can extend hospitalization, increase costs, and lead to secondary complications, making them more impactful on recovery than other minor complications. This distinction provides a clearer understanding of the perioperative safety profile of RATs versus VATs. Eight studies (RATs: 854; VATs: 1,059) reported postoperative pulmonary infections (Fig. 2d). The incidence was significantly lower in the RATs group, with patients experiencing about a 60% reduction in risk compared with VATs (OR = 0.39; 95% CI: 0.23–0.67; *P* = 0.0006, I² = 20%) [4, 23, 26, 33, 34, 36, 42, 49].

### Surgical duration

**Fig. 3:**
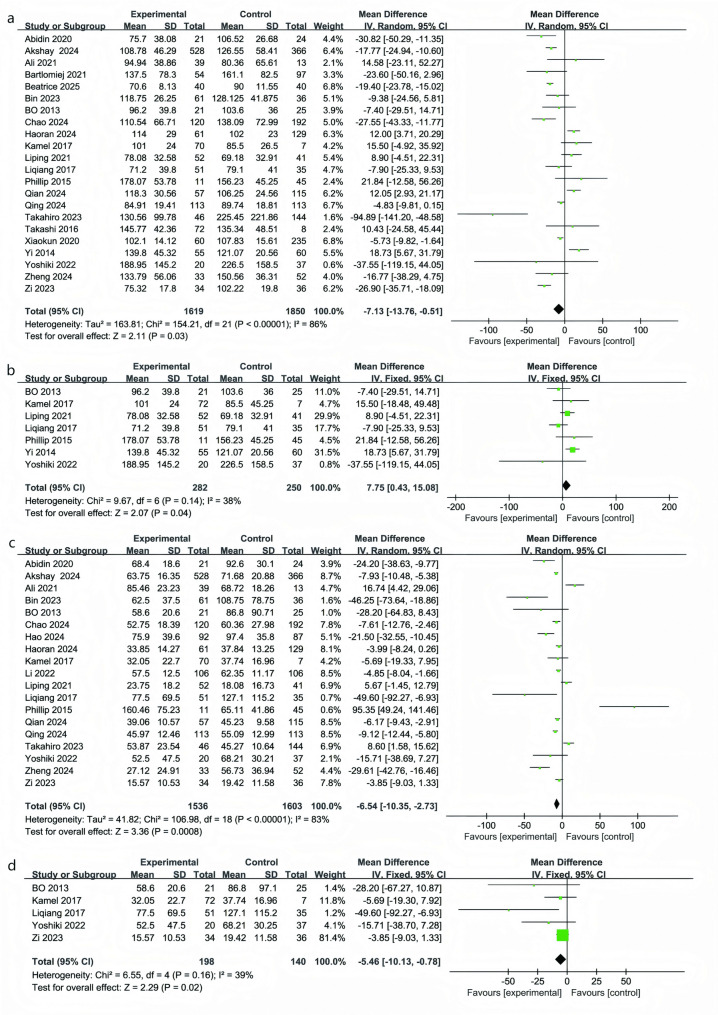
Forest plots comparing surgical duration (panels** a**-**b**) and intraoperative blood loss (panels **c**-**d**) between RATs and VATs, including subgroup analysis of the lateral approach

A meta-analysis of 22 studies (RATs: 1,619; VATs: 1,850) (Fig. 3a) showed that the average operative time was about 7 min shorter in the RATs group compared with VATs (MD = − 7.13 min; 95% CI: − 13.76 to − 0.51 min; *P* = 0.03) [4, 22–28, 34, 35, 37, 38, 40–49]. Although statistically significant, this time saving was relatively modest and should be interpreted in light of substantial between-study heterogeneity (I² = 86%), which likely reflects differences in surgical approach, case complexity, and surgeon experience.

To further explore heterogeneity, we performed a subgroup analysis restricted to studies that used a full lateral approach. Across seven studies (532 patients; RATs: 282, VATs: 250), the mean operative time was instead longer in the RATs group, by approximately 8 min on average (MD = 7.75 min; 95% CI: 0.43–15.08; *P* = 0.04, I² = 38%) (Fig. 3b) [27, 35, 37, 38, 40, 46, 47]. For procedures performed through a lateral approach, RATs tended to require more time, possibly reflecting the technical demands of precise dissection and the influence of the surgeon’s learning curve.

### Intraoperative blood loss

A meta-analysis of 19 studies (3,139 patients) showed that intraoperative blood loss was lower with RATs than with VATs (MD = − 6.50 mL; 95% CI − 10.35 to − 2.73; *P* = 0.0008; I² = 83%) (Fig. 3c) [4, 23, 24, 26–28, 33–38, 40–43, 47–49]. Given the high heterogeneity, a random-effects model was used. Despite this variation, results consistently favored RATs, indicating a modest yet significant reduction in bleeding. Variations in hemostatic devices (e.g., ultrasonic scalpel, bipolar cautery) and surgical techniques likely contributed to the heterogeneity.

In the subgroup of five studies using the full lateral approach (RATs = 198; VATs = 140), blood loss remained lower with RATs (MD = − 5.46 mL; 95% CI − 10.13 to − 0.78; *P* = 0.02; I² = 39%) (Fig. 3d) [27, 35, 38, 47, 49]. Although the difference was smaller than in the overall analysis, the consistent trend supports a reliable advantage of RATs in reducing intraoperative bleeding.

### Chest tube duration

**Fig. 4:**
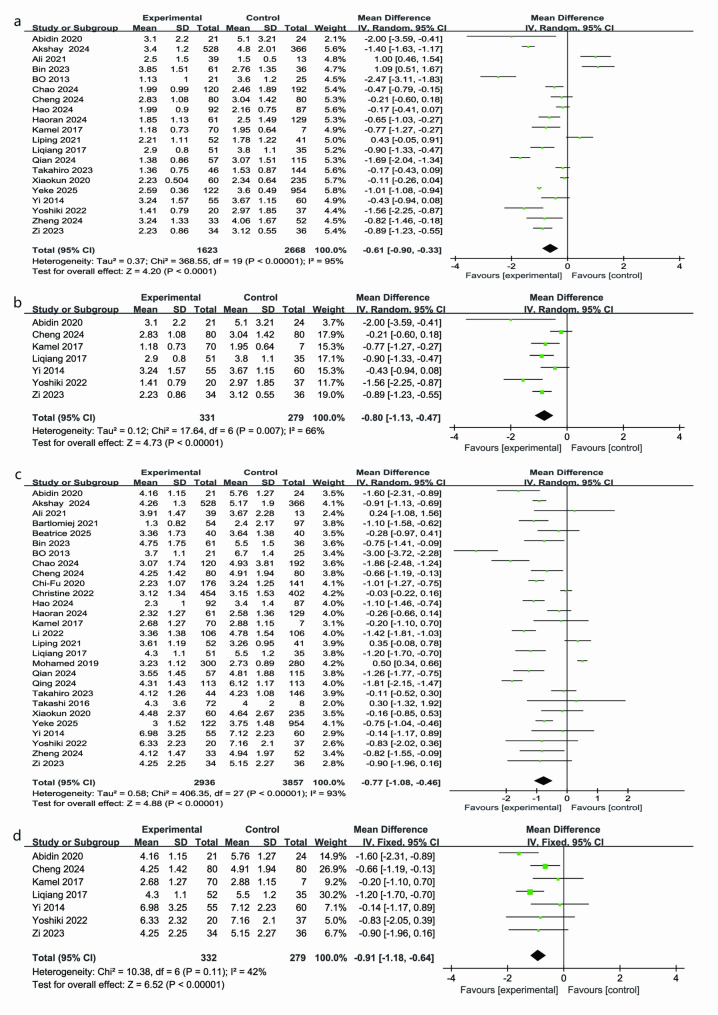
Forest plots comparing chest tube duration (panels **a**-**b**) and postoperative length of stay (panels **c**-**d**) between RATs and VATs, including subgroup analysis of the lateral approach Figure 5: Forest plots comparing total drainage volume (panel **a**) and the learning curve of the RATs group (panel **b**)

A pooled analysis of 20 studies (Fig. 4a) showed that the time to postoperative chest tube removal was on average 0.61 days shorter in the RATs group than in the VATs group (MD = − 0.61 days; 95% CI: − 0.90 to − 0.33 days; *P* < 0.05), though heterogeneity was high (I² = 95%) [4, 21, 23, 24, 26–29, 33–35, 37, 38, 41, 43, 45–49]. In the full lateral approach subgroup (7 studies; 331 patients in the RATs group and 279 in the VATs group) (Fig. 4b) showed similar significant reductions, chest tubes were removed 0.80 days earlier with robot assistance (MD = − 0.80 days; moderate between-study variability, I² = 66%; 95% confidence interval: − 1.13 to − 0.47 days; *P* < 0.05) [23, 29, 35, 38, 46, 47, 49].

### Postoperative length of stay

Meta-analysis of 28 studies (RATs: 2,936;VATs: 3,857) showed that postoperative length of stay was significantly shorter in the robotic group compared to the video-assisted group (MD = − 0.77 days; 95% CI: − 1.08 to − 0.46 days; *P* < 0.00001), despite very high heterogeneity (I² = 93%)(Fig. 4c) [4, 21–31, 33–49]. In the full lateral approach subgroup (7 studies; RATs:332; VATs:279), the robotic cohort had a significantly reduced hospital stay (MD = − 0.91 days; 95% CI: − 1.18 to − 0.64 days; *P* < 0.05) (Fig. 4d). Heterogeneity was moderate (I² = 42%; *P* = 0.11), and the constant-effects approach validated the stability of this result, indicating a clear advantage of robotic thymectomy in shortening postoperative hospitalization for lateral approaches [23, 29, 35, 38, 46, 47, 49].

### Total drainage volume

**Fig. 5:**
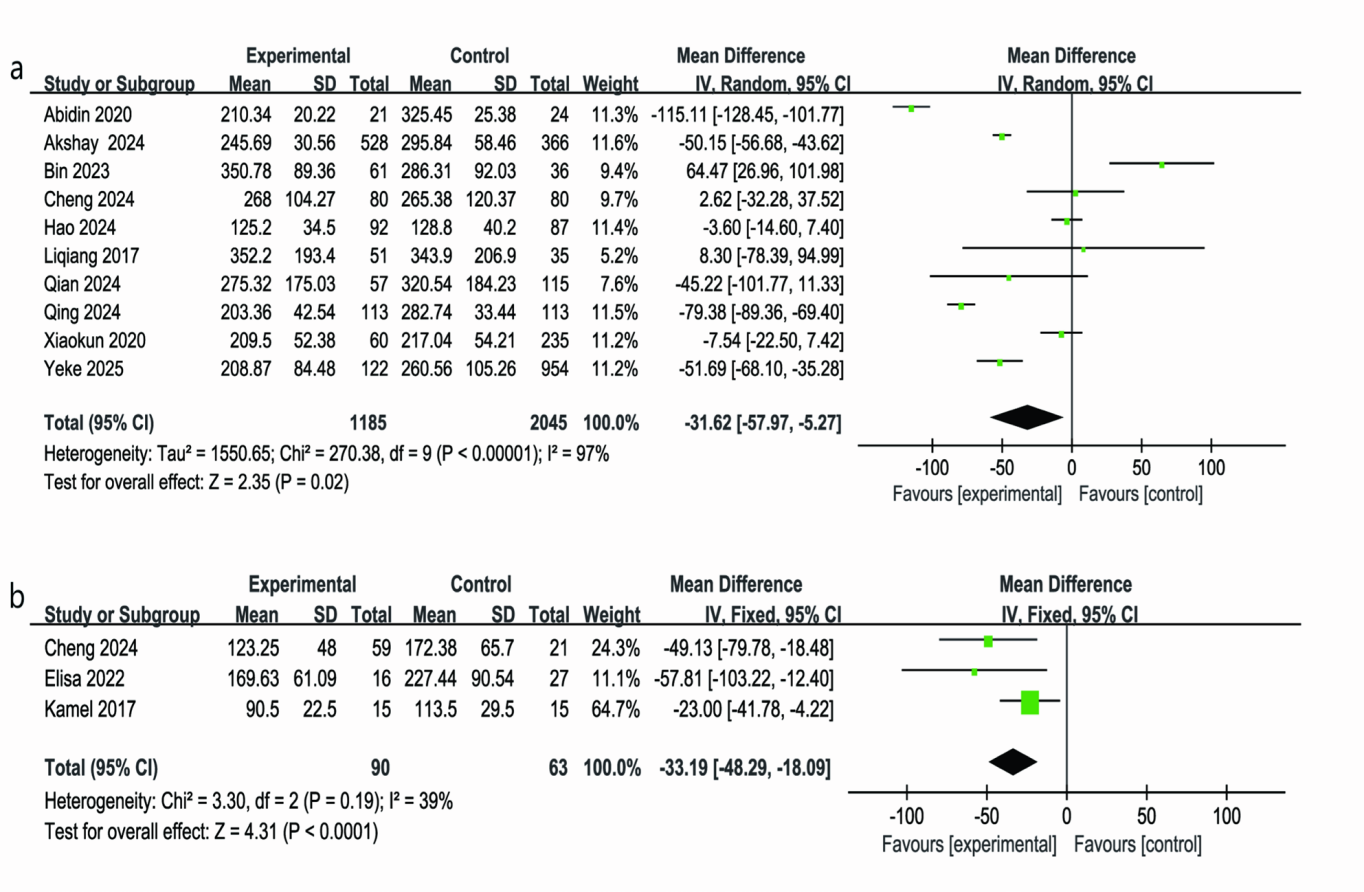
Forest plots comparing total drainage volume (panel **a**) and the learning curve of the RATs group (panel **b**)

Analysis of 10 studies (RATs: 1,185; VATs: 2,045) (Fig. 5a) showed that the total postoperative drainage volume was significantly lower in the RATs group compared to the VATs group (MD = − 31.62 mL; 95% CI: − 57.97 to − 5.27 mL; *P* = 0.02). However, marked differences were observed between the studies (I² = 97%) [4, 21, 23, 26, 29, 33, 38, 41, 42, 45], which may be attributed to factors such as differences in patient disease severity, resection extent, lymph node dissection, and drainage management, all of which could influence the total drainage volume. Despite these differences, the potential advantage of the RATs group in reducing total drainage volume remains noteworthy.

### Learning curve of the RATs

The learning curve for RATs was analyzed across 3 studies, including 153 patients [29, 35, 50]. The experimental group consisted of 90 cases at different stages of robotic surgery, while the control group included 63 corresponding reference cases. The analysis revealed that as surgical volume increased (from ≤ 20 cases in the early phase to >20 cases in the later phase), the operative time in the RATs group significantly decreased compared to the VATs group (MD = − 33.19 min; 95% CI: − 48.29 to − 18.09 min; *P* < 0.05). Moderate heterogeneity was observed between studies (I² = 39%; *P* = 0.19), and the constant-effects model confirmed the stability of the results (Fig. 5b). Although the control group contained 15 cases with a smaller sample size, their contribution was statistically balanced (e.g., Kamel 2017 accounted for 64.7%). Despite the smaller sample, the overall analysis still showed significant statistical differences, suggesting that the reduction in operative time with increased case experience reflects a reliable learning curve for RATs. These results reinforce the notion that RATs efficiency improves with surgeon experience, offering valuable insights for understanding the stages of proficiency in clinical practice.

### 30- and 90-day mortality

**Fig. 6:**
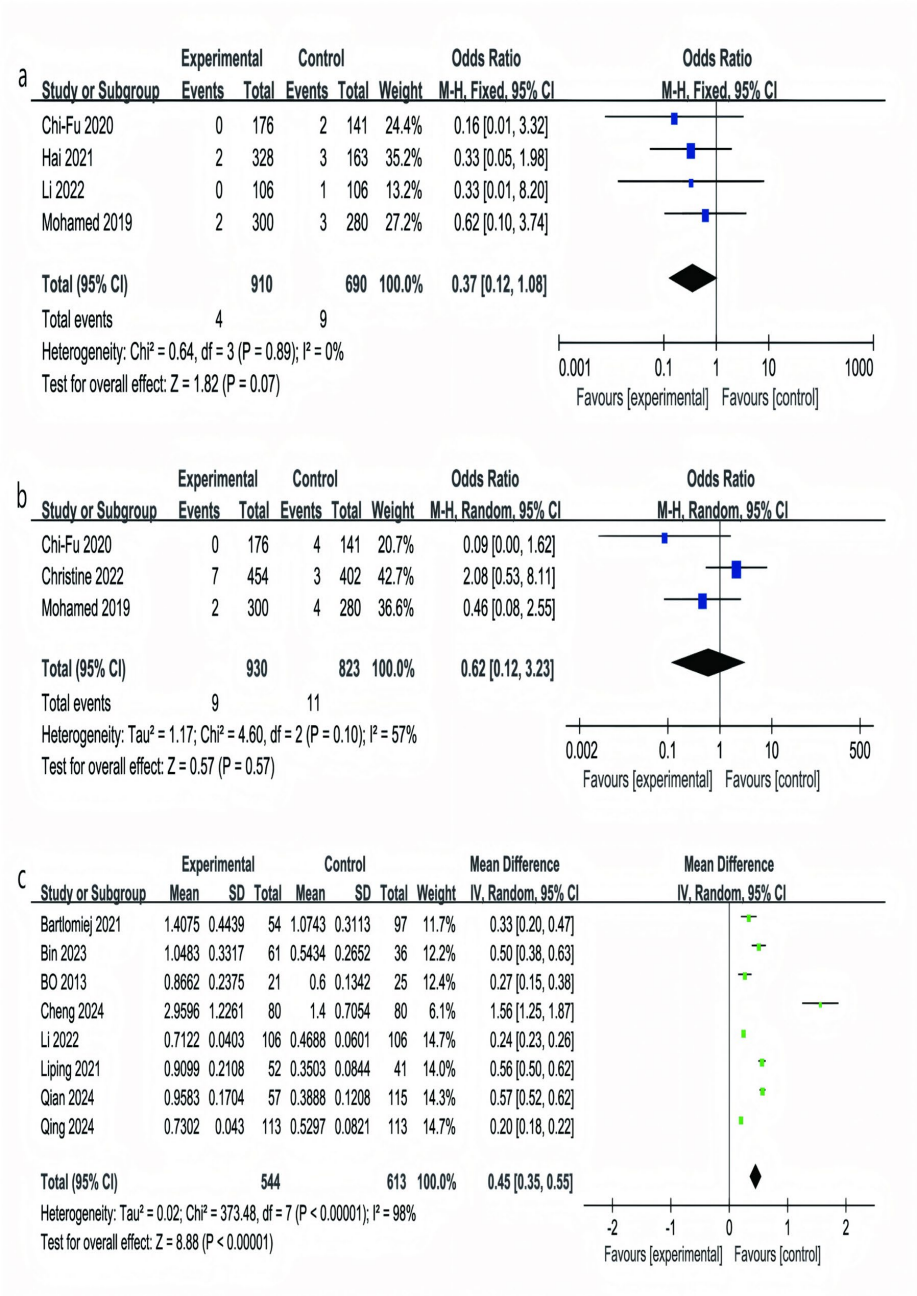
Forest plots comparing 30-day and 90-day mortality (panels **a**-**b**) and total hospitalization costs (panel **c**)

Figure 6a indicates that 30-day postoperative mortality occurred less often in RATs compared with VATs (OR = 0.37; 95% CI, 0.12–1.08; *P* = 0.07), showing little variability (I² = 0%; *P* = 0.89; constant-effects model), although the difference failed to reach a significant level in statistical testing [30, 32, 36, 39]. Analysis of 90-day mortality across three studies (RATs: 930;VATs: 823; Fig. 6b) revealed no significant difference (OR = 0.62; *P* = 0.57; CI:0.12–3.23), with moderate heterogeneity (I² = 57%; *P* = 0.10; random-effects model) [30, 31, 39]. Overall, these findings indicate comparable safety profiles for RATs and VATs at both 30 and 90 days postoperatively.

Total Hospitalization Costs.

As illustrated in Fig. 6c, pooled data from eight studies involving 1,157 patients (RATs: 544; VATs: 613) indicated that overall hospitalization expenses were markedly greater for RATs (MD = 0.45, representing the mean difference in cost, 95% CI: 0.35–0.55; *P* < 0.05). Substantial heterogeneity was observed (I² = 98%), so a random-effects model was applied. Individual study differences were notable (MD: 0.20–1.56), with Cheng 2024 reporting the largest excess cost (MD = 1.56) and Qing 2024 the smallest (MD = 0.20), indicating considerable variability in effect size across settings [25–27, 29, 36, 37, 41, 42]. Despite these variations, the overall trend of higher costs in the robot-assisted group remains consistent. The pronounced heterogeneity suggests that contextual factors—such as regional pricing structures, choice of consumables, length of stay, and patient comorbidities—likely modulate the magnitude of cost differences and should be accounted for when interpreting these findings and balancing cost versus clinical benefit in practice.

**Table 1:** Main characteristics of the included studies

Study ID (Author, Year)	Interventions	Sample size (*n*)		Gender (M\F)(*n*)	Age(Year) Mean ± SD	Median tumor size Mean ± SD(cm)	*Who histological classification* *(n)* A, AB, B1, B2, B3, Other						BMI(kg/m2) Mean ± SD	Approach (Right/Left)*n*	Myasthenia Gravis *n*(%)
Yeke 2025	RAT	1076	122	67\55	55.75 + 10.59	3.14 + 1.88	NA						NA	NA	6(4.92)
	VAT		954	431\523	52.78 + 13.85	3.06 + 1.54									11(1.15)
Akshay 2024	RAT	894	366	161\205	61 ± 12	4.9 ± 3.1	48	176	60	49	16	17	26.22 ± 5.30	NA	7 (2)
	VAT		528	295\233	60 ± 13	5.1 ± 2.8	92	229	75	71	42	19	27.36 ± 5.42		9 (2)
Bin 2023	RAT	97	61	63\34	46.10 ± 14.10	8.45 ± 1.45	13	15	13	12	8	0	23.01 ± 2.92	34/20	5 (8.20)
	VAT		36		47.60 ± 14.60	7.54 ± 0.96	3	10	5	9	4	5	22.98 ± 3.35	18/2	2 (5.56)
Cheng 2024	RAT	160	80	64\96	50.44 ± 13.40	4.10 ± 2.66	NA						25.14 ± 3.87	51/29	NA
	VAT		80		50.39 ± 12.43	3.71 ± 2.18							25.30 ± 3.32	51/29	
Yoshiki 2022	RAT	57	20	7\13	55.00 ± 27.00	3.73 ± 2.82	2	7	5	5	1	0	24.45 ± 6.75	16/4	4 (20.0)
	VAT		37	16\21	61.00 ± 26.00	3.95 ± 3.05	4	12	10	7	4	0	24.31 ± 8.33	28/9	11 (29.7)
Hai 2021	RAT	588	325	243\345	63.00 ± 34.00	4.52 ± 0.86	48	85	45	63	40	44	NA	NA	NA
	VAT		263		64.00 ± 34.00	5.12 ± 1.22	41	64	41	58	27	32			
Ali 2021	RAT	52	39	32\7	58 ± 20.5	4.82 ± 1.42	NA						NA	NA	NA
	VAT		13	9\4	68 ± 13.0	4.85 ± 0.85									
Abidin 2020	RAT	45	21	10\11	41.29 ± 7.05	3.22 ± 0.46	9	3	6	2	1	0	NA	19/2	4(19.4)
	VAT		24	8\16	42.52 ± 7.45	2.73 ± 0.57	8	4	5	6	1	0		24/0	5(20.8)
Xiaokun 2020	RAT	295	60	30\30	53.72 ± 13.11	5.50 ± 2.38	4	6	12	24	9	5	NA	NA	4(6)
	VAT		235	114\121	52.23 ± 12.56	6.05 ± 3.75	25	20	49	89	43	9			13(5.5)
Bartlomiej 2021	RAT	151	54	29\25	44.9 ± 15.8	NA	9	2	6	15	4	18	30.5 ± 8.3	NA	NA
	VAT		97	42\55	47.4 ± 15.2		8	21	19	12	20	17	32.4 ± 7.9		
Mohamed 2019	RAT	580	300	151\149	63.00 ± 9.00	4.72 ± 1.63	NA						NA	NA	NA
	VAT		280	149\131	62.00 ± 8.50	5.65 ± 2.15									
Liqiang 2017	RAT	86	51	21\30	48.8 ± 13.3	3.8 ± 1.1	10	14	12	11	2	2	NA	Full lateral approach	4 (7.8)
	VAT		35	19\16	50.3 ± 13.1	3.9 ± 1.1	4	15	7	5	3	1			1 (2.9)
Kamel 2017	RAT	77	70	23\47	NA	4.72 ± 1.23	NA						NA	Full lateral approach	19(27.1)
	VAT		7	5\2		4.29 ± 1.44									1(14.2)
Takashi 2016	RAT	80	72	NA	53.4 ± 14.8	3.2 ± 1.7	11	21	9	15	8	8	22.8 ± 3.7	NA	13 (18.1)
	VAT		8		55.5 ± 9.9	4.1 ± 2.1	2	1	2	3	0	0	23.8 ± 5.1		2 (25.0)
Phillip 2015	RAT	56	11	6\5	52.20 ± 25.50	NA	NA						29.02 ± 8.85	Full lateral approach	2(18.1)
	VAT		45	19\26	50.60 ± 32.00								33.82 ± 13.97		12(26.1)
BO 2013	RAT	46	21	9\12	52.7 ± 7.8	2.91 ± 0.77	NA						NA	Full lateral approach	NA
	VAT		25	13\12	53.4 ± 5.4	3.04 ± 0.79									
Yi 2014	RAT	115	55	25\30	41.38 ± 9.5	NA	NA						NA	Full lateral approach	NA
	VAT		60	28\32	43.53 ± 8.8										
Haoran 2024	RAT	190	61	28\31	58.00 ± 6.50	13.21 ± 3.52	4	22	7	13	3	12	23.92 ± 2.14	NA	5(8.2)
	VAT		129	66\63	56.00 ± 7.00	9.23 ± 3.77	10	51	19	18	6	25	24.46 ± 1.92		9(7.0)
Beatrice 2025	RAT	80	40	14\26	62.26 ± 8.57	6.14 ± 1.86	5	13	5	5	12	0	NA	NA	8 (20)
	VAT		40	15\25	61.28 ± 9.8	4.71 ± 0.48	8	14	7	8	3	0			7 (17.5)
Qian 2024	RAT	172	57	34\23	48 ± 13	4.22 ± 1.18	NA						24.83 ± 3.47	NA	NA
	VAT		115	67\48	43 ± 14	3.47 ± 0.98							25.33 ± 2.97		
Hao 2024	RAT	179	92	47\45	50.4 ± 13.5	4.4 ± 1.9	NA						NA	NA	NA
	VAT		87	39\48	51.5 ± 14.2	4.5 ± 2.2									
Qing 2024	RAT	226	113	51\62	50.91 ± 5.24	3.27 ± 0.62	NA						24.31 ± 1.53	NA	NA
	VAT		113	59\54	50.41 ± 2.67	3.24 ± 0.60							24.15 ± 1.46		
Zheng 2024	RAT	85	33	16\17	53.91 ± 13.32	8.74 ± 2.39	3	7	1	7	4	11	NA	NA	3 (9.09)
	VAT		52	21\31	51.73 ± 13.23	7.93 ± 1.97	6	10	6	7	8	15			5 (9.62)
Chao 2024	RAT	312	120	61\59	51.62 ± 13.70	5.44 ± 2.87	NA						24.18 ± 4.13	NA	10 (8.3)
	VAT		192	70\122	53.36 ± 13.80	4.36 ± 2.65							24.37 ± 3.48		17 (8.9)
Li 2022	RAT	212	106	122\90	45.38 ± 11.63	4.91 ± 1.44	NA						24.90 ± 2.15	NA	NA
	VAT		106		47.88 ± 8.13	5.23 ± 1.78							24.77 ± 2.45		
Takahiro 2023	RAT	190	46	28\18	53.00 ± 25.00	NA	NA						NA	24/14	NA
	VAT		144	84\64	46.50 ± 36.50									56/51	
Zi 2023	RAT	70	34	14\20	41.03 ± 7.38	3.70 ± 1.16	NA						23.41 ± 4.12	Full lateral approach	NA
	VAT		36	17\19	43.06 ± 6.82	4.07 ± 1.20							23.28 ± 3.75		
Christine 2022	RAT	856	454	223\231	62.50 ± 7.50	4.85 ± 1.65	NA						NA	NA	NA
	VAT		402	196\206	61.00 ± 10.00	5.65 ± 2.15									
Liping 2021	RAT	93	52	27\25	49.00 ± 27.00	4.75 ± 3.95	NA						NA	Full lateral approach	4 (7.7)
	VAT		41	18\23	48.50 ± 22.50	4.00 ± 3.20									0 (0.0)
Chi-Fu 2020	RAT	317	176	117\59	59 ± 12.36	4.9 ± 1.4	20	43	27	39	14	33	NA	NA	NA
	VAT		141	66\75	62 ± 9.56	5.7 ± 2.1	14	35	24	26	21	21			

**Table 2:** Results from Thirty studies

Study ID (Author, Year)	Interventions	Conversion to thoracotomy, *n*(%)	Operative time (min)	R0 resection *n*(%)	intraoperative blood loss Mean ± SD(mL)	Chest tube placement time Mean ± SD(Days)	Total drainage volume (mL)	Postoperative stay Mean ± SD(Days)	Total complications *n*(%)	Arrhythmia *n*(%)	Total in-hospital costs Mean ± SD($)	Pulmonary infection *n*(%)	30 days mortality *n*(%)	90 days mortality *n*(%)
Yeke 2025	RAT	NA	NA	NA	NA	2.59 ± 0.36	208.87 ± 84.48	3 ± 1.52	NA	NA	NA	NA	NA	NA
	VAT					3.6 ± 0.49	260.56 ± 105.26	3.75 ± 1.48						
Akshay 2024	RAT	NA	108.78 ± 46.29	NA	63.75 ± 6.35	3.4 ± 1.2	245.69 ± 30.56	4.26 ± 1.3	NA	NA	NA	2(0.5)	NA	NA
	VAT		126.55 ± 58.41		71.68 ± 20.88	4.8 ± 2.01	295.84 ± 58.46	5.17 ± 1.9				20(3.8)		
Bin 2023	RAT	NA	118.75 ± 26.25	58 (95.08)	62.50 ± 37.50	3.85 ± 1.51	350.78 ± 89.36	4.75 ± 1.75	NA	NA	10,483.63 ± 3,317.86	2(3.28)	NA	NA
	VAT		128.125 ± 41.87	34 (94.44)	108.75 ± 78.75	2.76 ± 1.35	286.31 ± 92.03	5.5 ± 1.5			5434.00 ± 2,652.80	1(2.78)		
Cheng 2024	RAT	1 (1.3)	NA	NA	NA	2.83 ± 1.08	268.00 ± 104.27	4.25 ± 1.42	NA	NA	29,596.96 ± 12,260.85	NA	0(0)	NA
	VAT	0				3.04 ± 1.42	265.38 ± 120.30	4.91 ± 1.94			14,097.24 ± 7,054.00		0(0)	
Yoshiki 2022	RAT	NA	188.95 ± 145.2	NA	52.50 ± 47.50	1.41 ± 0.79	NA	6.33 ± 2.32	4 (20.0)	0(0)	NA	NA	NA	NA
	VAT		226.5 ± 158.5		68.21 ± 30.25	2.97 ± 1.85		7.16 ± 2.10	8 (21.6)	1 (2.7)				
Hai 2021	RAT	17 (5.0)	NA	12(71.4)	NA	NA	NA	NA	NA	NA	NA	NA	2 (0.6)	NA
	VAT	41 (13.4)		25(61.3)									3 (1.1)	
Ali 2021	RAT	NA	94.94 ± 38.86	35 (89.7)	85.46 ± 23.23	2.5 ± 1.5	NA	3.91 ± 1.47	NA	NA	NA	NA	NA	NA
	VAT		80.36 ± 65.61	13 (100.0)	68.72 ± 18.26	1.50 ± 0.5		3.67 ± 2.28						
Abidin 2020	RAT	NA	75.70 ± 38.08	NA	68.4 ± 18.6	3.10 ± 2.2	210.34 ± 20.22	4.16 ± 1.15	2(14.2)	NA	NA	1(4.76)	0(0)	NA
	VAT		106.52 ± 26.68		92.6 ± 30.1	5.10 ± 3.21	325.45 ± 25.38	5.76 ± 1.27	3(12.5)			0(0)	0(0)	
Xiaokun 2020	RAT	1(1.66)	102.10 ± 14.12	NA	NA	2.23 ± 0.50	209.50 ± 52.38	4.48 ± 2.37	2(3.33)	NA	NA	NA	NA	NA
	VAT	8(3.40)	107.83 ± 15.61			2.34 ± 0.64	217.04 ± 54.21	4.64 ± 2.67	10(4.26)					
Bartlomiej 2021	RAT	NA	187.5 ± 78.3	NA	NA	NA	NA	1.3 ± 0.82	NA	0(0)	14,075 ± 4,439	NA	NA	NA
	VAT		161.1 ± 82.5					2.4 ± 2.17		5 (5)	10,743 ± 3,113			
Mohamed 2019	RAT	17 (6)	NA	236 (77)	NA	NA	NA	3.23 ± 1.12	NA	NA	NA	NA	2 (1)	2 (1)
	VAT	37 (13)		204 (70)				2.73 ± 0.89					3 (1.5)	4 (2)
Liqiang 2017	RAT	NA	71.2 ± 39.8	NA	77.5 ± 69.5	2.9 ± 0.8	352.2 ± 193.4	4.3 (1.1)	NA	NA	NA	NA	NA	NA
	VAT		79.1 ± 41.0		127.1 ± 115.2	3.8 ± 1.1	343.9 ± 206.9	5.5 (1.2)						
Kamel 2017	RAT	5(7)	101 ± 24	NA	32.05 ± 22.70	1.18 ± 0.73	NA	2.68 ± 1.27	NA	NA	NA	NA	NA	NA
	VAT	0(0)	85.50 ± 26.50		37.74 ± 16.96	1.95 ± 0.64		2.88 ± 1.15						
Takashi 2016	RAT	NA	145.77 ± 42.36	NA	NA	NA	NA	4.3 ± 3.6	NA	1(1.39)	NA	NA	NA	NA
	VAT		135.34 ± 48.51					4.0 ± 2.0		0(0)				
Phillip 2015	RAT	0	178.07 ± 53.78	NA	160.46 ± 75.23	NA	NA	NA	NA	2(18.1)	NA	NA	NA	NA
	VAT	0	156.23 ± 45.25		65.11 ± 41.86					0(0)				
BO 2013	RAT	0	96.2 ± 39.8	NA	58.6 ± 20.6	1.13 ± 1	NA	3.7 ± 1.1	NA	NA	8,662 ± 2,375	NA	NA	NA
	VAT	1(4)	103.6 ± 36		86.8 ± 97.1	3.6 ± 1.2		6.7 ± 1.4			6,097 ± 1,342			
Yi 2014	RAT	NA	139.80 ± 45.32	NA	NA	3.24 ± 1.57	NA	6.98 ± 3.25	NA	NA	NA	NA	NA	NA
	VAT		121.07 ± 20.56			3.67 ± 1.15		7.12 ± 2.23						
Haoran 2024	RAT	2(3.3)	114 ± 29	NA	33.85 ± 14.27	1.85 ± 1.13	NA	2.32 ± 1.27	0(0)	0	NA	0(0)	NA	NA
	VAT	3(2.3)	102 ± 23		37.84 ± 13.25	2.5 ± 1.49		2.58 ± 1.36	4(3.1)	3 (2.3)		1(0.77)		
Beatrice 2025	RAT	0 (0)	70.60 ± 8.13	39 (97.5)	NA	NA	NA	3.36 ± 1.73	NA	NA	NA	NA	NA	NA
	VAT	6 (15)	90 ± 11.55	39 (97.5)				3.64 ± 1.38						
Qian 2024	RAT	0(0)	118.30 ± 30.56	57(100)	39.06 ± 10.57	1.38 ± 0.86	275.32 ± 175.03	3.55 ± 1.45	NA	NA	9,583.78 ± 1,704.49	NA	NA	NA
	VAT	4 (3.5)	106.25 ± 24.56	113(98.3)	45.23 ± 9.58	3.07 ± 1.51	320.54 ± 184.23	4.81 ± 1.88			3,888.57 ± 1,208.42			
Hao 2024	RAT	NA	NA	NA	75.9 ± 39.6	1.99 ± 0.9	125.2 ± 34.5	2.3 ± 1.0	NA	NA	NA	5(5.4)	NA	NA
	VAT				97.4 ± 35.8	2.16 ± 0.75	128.8 ± 40.2	3.4 ± 1.4				7(8.0)		
Qing 2024	RAT	NA	84.91 ± 19.41	NA	45.97 ± 12.46	NA	203.36 ± 42.54	4.31 ± 1.43	NA	2(1.8)	7,302.17 ± 430.47	4(3.5)	NA	NA
	VAT		89.74 ± 18.81		55.09 ± 12.99		282.74 ± 33.44	6.12 ± 1.17		3(2.7)	5,297.98 ± 821.25	6(5.3)		
Zheng 2024	RAT	NA	133.79 ± 56.06	NA	27.12 ± 24.91	3.24 ± 1.33	NA	4.12 ± 1.47	NA	NA	NA	NA	NA	NA
	VAT		150.56 ± 36.31		56.73 ± 36.94	4.06 ± 1.67		4.94 ± 1.97						
Chao 2024	RAT	1 (0.8)	220.39 ± 66.71	97 (94.2)	52.75 ± 18.39	1.99 ± 0.99	NA	3.07 ± 1.74	0	NA	NA	NA	NA	NA
	VAT	1 (0.5)	138.09 ± 72.99	98 (98)	60.36 ± 27.98	2.46 ± 1.89		3.81 ± 4.93	2 (1)					
Li 2022	RAT	0(0)	NA	NA	57.50 ± 12.50	NA	NA	3.36 ± 1.38	6 (5.7)	1 (0.9)	7,122.22 ± 403.56	3 (2.8)	0	NA
	VAT	0(0)			62.35 ± 11.17			4.78 ± 1.54	15 (14.2)	2 (1.9)	4,688.54 ± 601.71	10 (9.4)	1 (0.9)	
Takahiro 2023	RAT	0(0)	130.56 ± 99.78	NA	53.87 ± 23.54	1.36 ± 0.75	NA	4.12 ± 1.26	1 (2.2)	NA	NA	NA	NA	NA
	VAT	1 (0.7)	225.45 ± 221.86		45.27 ± 10.64	1.53 ± 0.87		4.23 ± 1.08	8 (5.6)					
Zi 2023	RAT	0	75.32 ± 17.80	NA	15.57 ± 10.53	2.23 ± 0.86	NA	4.25 ± 2.25	2 (5.9)	NA	NA	1 (2.9)	NA	NA
	VAT	1 (2.8)	102.22 ± 19.80		19.42 ± 11.58	3.12 ± 0.55		5.15 ± 2.27	3 (8.3)			0(0)		
Christine 2022	RAT	22 (4.9)	NA	NA	NA	NA	NA	3.12 ± 1.34	NA	NA	NA	NA	NA	7 (2.3)
	VAT	59 (14.7)						3.15 ± 1.53						3 (1.0)
Liping 2021	RAT	0(0)	78.08 ± 32.58	NA	23.75 ± 18.20	2.21 ± 1.11	NA	3.61 ± 1.19	3(5.9)	1 (1.9)	9,099.81 ± 2,168.02	NA	NA	NA
	VAT	2 (5.0)	69.18 ± 32.91		18.08 ± 16.73	1.78 ± 1.22		3.26 ± 0.95	3(7.4)	0	3,503.03 ± 844.66			
Chi-Fu 2020	RAT	8(4.55)	NA	131 (74.4)	NA	NA	NA	2.23 ± 1.07	NA	NA	NA	NA	0(0)	0(0)
	VAT	26 (18.4)		98 (69.5)				3.24 ± 1.25					2(1.1)	4(2.8)

**Table 3:** Risk of bias assessment

Study ID	Selection	Comparability	Outcome	Total
Yeke 2025	✩✩✩✩	✩	✩✩✩	✩✩✩✩✩✩✩✩
Akshay 2024	✩✩✩✩	✩	✩✩✩	✩✩✩✩✩✩✩✩
Bin 2023	✩✩✩✩	✩	✩✩✩	✩✩✩✩✩✩✩✩
Cheng 2024	✩✩✩✩	✩	✩✩✩	✩✩✩✩✩✩✩✩
Yoshiki 2022	✩✩✩✩	✩	✩✩✩	✩✩✩✩✩✩✩✩
Hai 2021	✩✩✩✩	✩	✩✩	✩✩✩✩✩✩✩
Ali 2021	✩✩✩✩	✩	✩✩✩	✩✩✩✩✩✩✩✩
Abidin 2020	✩✩✩✩	✩	✩✩✩	✩✩✩✩✩✩✩✩
Xiaokun 2020	✩✩✩✩	✩	✩✩	✩✩✩✩✩✩✩
Bartlomiej 2021	✩✩✩✩	✩	✩✩✩	✩✩✩✩✩✩✩✩
Mohamed 2019	✩✩✩✩	✩	✩✩✩	✩✩✩✩✩✩✩✩
Liqiang 2017	✩✩✩✩	✩	✩✩✩	✩✩✩✩✩✩✩✩
Kamel 2017	✩✩✩✩	✩	✩✩✩	✩✩✩✩✩✩✩✩
Takashi 2016	✩✩✩✩	✩	✩✩	✩✩✩✩✩✩✩
Phillip 2015	✩✩✩✩	✩	✩✩✩	✩✩✩✩✩✩✩✩
BO 2013	✩✩✩✩	✩	✩✩✩	✩✩✩✩✩✩✩✩
Yi 2014	✩✩✩✩	✩	✩✩✩	✩✩✩✩✩✩✩✩
Haoran 2024	✩✩✩✩	✩	✩✩✩	✩✩✩✩✩✩✩✩
Beatrice 2025	✩✩✩✩	✩	✩✩✩	✩✩✩✩✩✩✩✩
Qian 2024	✩✩✩✩	✩	✩✩✩	✩✩✩✩✩✩✩✩
Hao 2024	✩✩✩✩	✩	✩✩	✩✩✩✩✩✩✩
Qing 2024	✩✩✩✩	✩	✩✩✩	✩✩✩✩✩✩✩✩
Zheng 2024	✩✩✩✩	✩	✩✩✩	✩✩✩✩✩✩✩✩
Chao 2024	✩✩✩✩	✩	✩✩✩	✩✩✩✩✩✩✩✩
Li 2022	✩✩✩✩	✩	✩✩✩	✩✩✩✩✩✩✩✩
Takahiro 2023	✩✩✩✩	✩	✩✩✩	✩✩✩✩✩✩✩✩
Zi 2023	✩✩✩✩	✩	✩✩✩	✩✩✩✩✩✩✩✩
Christine 2022	✩✩✩✩	✩	✩✩	✩✩✩✩✩✩✩
Liping 2021	✩✩✩✩	✩	✩✩	✩✩✩✩✩✩✩
Chi-Fu 2020	✩✩✩✩	✩	✩✩✩	✩✩✩✩✩✩✩✩

**Table 4:** Evaluation of the risk of bias in the studies included in the analysis through the use of the ROBINS-I tool

Study ID	Bias due to confounding	Bias in selection of participants	Bias in classification of interventions	Bias due to deviations from intended interventions	Bias due to missing data	Bias in measurement of outcomes	Bias in selection of the reported result	Overall risk of bias
Yeke 2025	Moderate	Moderate	Low	Low	Low	Low	Moderate	Moderate
Akshay 2024	Moderate	Low	Low	Low	Low	Low	Moderate	Moderate
Bin 2023	Moderate	Low	Low	Low	Low	Low	Moderate	Moderate
Cheng 2024	Moderate	Low	Low	Low	Low	Moderate	Low	Moderate
Yoshiki 2022	Moderate	Low	Low	Low	Low	Low	Moderate	Moderate
Hai 2021	Moderate	Low	Low	Low	Low	Moderate	Moderate	Moderate
Ali 2021	Moderate	Moderate	Low	Low	Low	Low	Moderate	Moderate
Abidin 2020	Moderate	Low	Low	Low	Low	Low	Moderate	Moderate
Xiaokun 2020	Moderate	Low	Low	Low	Low	Moderate	Low	Moderate
Bartlomiej 2021	Moderate	Low	Low	Low	Low	Low	Moderate	Moderate
Mohamed 2019	Moderate	Low	Low	Low	Low	Moderate	Moderate	Moderate
Liqiang 2017	Moderate	Moderate	Low	Low	Low	Low	Moderate	Moderate
Kamel 2017	Moderate	Low	Low	Low	Low	Low	Moderate	Moderate
Takashi 2016	Moderate	Low	Low	Low	Low	Moderate	Low	Moderate
Phillip 2015	Moderate	Low	Low	Low	Low	Low	Moderate	Moderate
BO 2013	Moderate	Low	Low	Low	Low	Moderate	Moderate	Moderate
Yi 2014	Moderate	Moderate	Low	Low	Low	Low	Moderate	Moderate
Haoran 2024	Moderate	Low	Low	Low	Low	Moderate	Low	Moderate
Beatrice 2025	Moderate	Low	Low	Low	Low	Low	Moderate	Moderate
Qian 2024	Moderate	Low	Low	Low	Low	Moderate	Low	Moderate
Hao 2024	Moderate	Low	Low	Low	Low	Low	Moderate	Moderate
Qing 2024	Moderate	Low	Low	Low	Low	Moderate	Low	Moderate
Zheng 2024	Moderate	Low	Low	Low	Low	Moderate	Moderate	Moderate
Chao 2024	Moderate	Moderate	Low	Low	Low	Low	Moderate	Moderate
Li 2022	Moderate	Low	Low	Low	Low	Moderate	Low	Moderate
Takahiro 2023	Moderate	Low	Low	Low	Low	Low	Moderate	Moderate
Zi 2023	Moderate	Low	Low	Low	Low	Moderate	Moderate	Moderate
Christine 2022	Moderate	Moderate	Low	Low	Low	Low	Moderate	Moderate
Liping 2021	Moderate	Moderate	Low	Low	Low	Low	Moderate	Moderate
Chi-Fu 2020	Moderate	Low	Low	Low	Low	Moderate	Low	Moderate

## Discussion

This systematic review and meta-analysis included a comprehensive evidence base (30 articles, 7,347 patients), providing stronger evidence compared to earlier reviews that were limited by smaller sample sizes and focused on selective perioperative outcomes. Our study expanded the scope by including key endpoints such as R0 resection rates, hospitalization costs, and the learning curve for RATs [51–53]. This study followed PRISMA guidelines, was prospectively registered in PROSPERO, and incorporated bias and sensitivity analyses to enhance result reliability. These methods support a balanced evaluation of robotic surgery and may inform future clinical research.

Our findings highlight the clear advantages of RATs over VATs in key clinical outcomes. Specifically, RATs reduced the risk of conversion to open surgery by 66% (OR = 0.34; *P* < 0.05), increased R0 resection rates by 65% (OR = 1.65; *P* = 0.01), decreased overall complications by 47% (OR = 0.53; *P* = 0.02), and reduced postoperative pulmonary infections by 61% (OR = 0.39; *P* = 0.0006). These improvements are attributed to the enhanced three-dimensional visualization, tremor filtration, and increased instrument flexibility provided by the robotic platform, which together improve precision and safety during thymoma resection. The differences in blood loss (MD = − 6.5 mL) and drainage volume (MD = − 31.6 mL) were small and may have limited clinical relevance, though they might contribute modestly to postoperative recovery and potentially lower the risk of postoperative complications. Given the challenges of measuring these small changes, we suggest that their clinical impact should be considered in the broader context of recovery and patient outcomes. Furthermore, RATs was associated with shorter chest tube duration and postoperative length of stay, particularly for full lateral-approach procedures [8], although these differences may seem small, they are clinically relevant as they can lead to reduced in-hospital stay and overall healthcare costs, particularly in the context of rapid recovery and minimizing hospital resource utilization. These findings highlight the broader potential benefits of RATs, despite the seemingly modest magnitude of differences.

A focused analysis of the RATs learning curve showed a significant reduction in operative time by 33 min once surgeons surpassed 20 cases (MD = − 33.19 min; *P* < 0.05). This finding aligns with previous reports that proficiency is achieved after approximately 25 robotic thymectomy procedures, emphasizing the importance of structured training programs to facilitate the safe adoption of RATs. Moreover, RATs demonstrates clear advantages in larger thymomas (≥ 5 cm), where precise dissection and 3D visualization are crucial for safe resection, particularly when tumors are closely adhered to critical structures [54, 55]. Although RATs incurred higher hospitalization costs, the high heterogeneity suggests that regional pricing and healthcare factors strongly influence these outcomes. Standardized cost-effectiveness analyses are required to clarify economic implications.

Another important consideration is the evolution of robotic platforms. While the Da Vinci system is commonly used for thymectomy with both multi-portal and single-port approaches, newer platforms may offer greater flexibility, enhanced visualization, and reduced invasiveness, potentially improving outcomes, particularly for complex or large tumors. Additionally, combining RATs with neoadjuvant therapy, especially for larger or advanced tumors, may improve outcomes by reducing tumor size and enhancing respectability [56], leading to higher R0 resection rates and lower recurrence risks. Future studies comparing the Da Vinci system with newer robotic platforms, as well as exploring RATs in conjunction with neoadjuvant therapy, are crucial for fully assessing the potential of robotic surgery in thymoma resection.

### Limitations and future directions

Despite adherence to PRISMA and PICOS frameworks, several limitations should be acknowledged. Mortality analyses were underpowered because of few events. Second, high heterogeneity in hospitalization costs and drainage outcomes underscores the need for standardized reporting of economic and perioperative data. Third, most of the included studies were non-randomized, and residual confounding related to surgeon experience, institutional protocols, and patient selection cannot be excluded. Given regional differences in healthcare systems and insurance policies, country-specific analyses may be more informative than global pooling. The learning curve for RATs should also be interpreted cautiously, particularly for large (≥ 5 cm) or complex tumors, where size and stage can influence surgical proficiency. In addition, variation in surgical approaches—often not clearly distinguished as unilateral or bilateral—limits the feasibility of further stratified analysis. Finally, few studies reported long-term outcomes, and inconsistent statistical methods prevented reliable synthesis of 3- and 5-year survival data.

## Conclusion

This meta-analysis compared perioperative outcomes between RATs and VATs for thymoma. RATs was associated with fewer complications, shorter recovery, and higher R0 resection rates in the included studies. Although it involves a steeper learning curve and higher costs, its potential benefits warrant further confirmation through prospective randomized trials. Additionally, its potential to reduce postoperative resource utilization and improve patient outcomes supports the continued application of RATs. Future high-quality randomized trials are needed to optimize patient selection, refine economic models, and set standardized training benchmarks for broader clinical adoption.

## Supplementary Information

Supplementary Material 1

Supplementary Material 2

## Data Availability

The datasets generated and/or analyzed during the present study are available from the corresponding author upon reasonable request.

## References

[1] Du X, et al. Risk factor analysis of thymoma resection and its value in guiding clinical treatment. Cancer Med. 2023;12(12):13408–14. 37156630 10.1002/cam4.6043 PMC10315718

[2] Muto Y, Okuma Y. Therapeutic options in thymomas and thymic carcinomas. Expert Rev Anticancer Ther. 2022;22(4):401–13. 35266421 10.1080/14737140.2022.2052278

[3] Comacchio GM, et al. Robotic thymectomy in thymic tumours: a multicentre, nation-wide study. Eur J Cardiothorac Surg. 2024. 10.1093/ejcts/ezae178. 38663851 10.1093/ejcts/ezae178

[4] Patel AJ, et al. Robotic-assisted versus video-assisted thoracoscopic surgery for thymic epithelial tumours, from the European Society of Thoracic Surgeons Database. Eur J Cardiothorac Surg. 2024. 10.1093/ejcts/ezae346. 39325852 10.1093/ejcts/ezae346

[5] Yin X, et al. Expert consensus on subxiphoid and subcostal arch thoracoscopic resection for the treatment of thymoma. Thorac Cancer. 2025;16(12):e70094. 40568789 10.1111/1759-7714.70094 PMC12199056

[6] Marulli G, et al. Robot-aided thoracoscopic thymectomy for early-stage thymoma: a multicenter European study. J Thorac Cardiovasc Surg. 2012;144(5):1125–30. 22944082 10.1016/j.jtcvs.2012.07.082

[7] Khan AZ, et al. Robotic thoracic surgery in inflammatory and infective diseases. Ann Cardiothorac Surg. 2019;8(2):241–9. 31032208 10.21037/acs.2019.02.05 PMC6462553

[8] Lee JH, et al. Subxiphoid single-port robotic thymectomy using the single-port robotic system versus VATS: a multi-institutional, retrospective, and propensity score-matched study. Cancers (Basel). 2024. 10.3390/cancers16162856. 39199627 10.3390/cancers16162856 PMC11353098

[9] Chatterjee S, et al. Advancements in robotic surgery: innovations, challenges and future prospects. J Robot Surg. 2024;18(1):28. 38231455 10.1007/s11701-023-01801-w

[10] Battaglia E, et al. Rethinking autonomous surgery: focusing on enhancement over autonomy. Eur Urol Focus. 2021;7(4):696–705. 34246619 10.1016/j.euf.2021.06.009 PMC10394949

[11] He T, et al. Postoperative radiotherapy for completely resected thymoma and thymic carcinoma: a systematic review and meta-analysis. PLoS One. 2024;19(8):e0308111. 39213310 10.1371/journal.pone.0308111 PMC11364254

[12] O’Sullivan KE, et al. A systematic review of robotic versus open and video assisted thoracoscopic surgery (VATS) approaches for thymectomy. Ann Cardiothorac Surg. 2019;8(2):174–93. 31032201 10.21037/acs.2019.02.04 PMC6462547

[13] Page MJ, et al. PRISMA 2020 explanation and elaboration: updated guidance and exemplars for reporting systematic reviews. BMJ. 2021;372:n160. 33781993 10.1136/bmj.n160 PMC8005925

[14] Amir-Behghadami M, Janati A. Population, intervention, comparison, outcomes and study (PICOS) design as a framework to formulate eligibility criteria in systematic reviews. Emerg Med J. 2020;37(6):387. 32253195 10.1136/emermed-2020-209567

[15] Moreau D, Gamble B. Conducting a meta-analysis in the age of open science: tools, tips, and practical recommendations. Psychol Methods. 2022;27(3):426–32. 32914999 10.1037/met0000351

[16] Kwon D, Reis IM. Simulation-based estimation of mean and standard deviation for meta-analysis via approximate bayesian computation (ABC). BMC Med Res Methodol. 2015;15:61. 26264850 10.1186/s12874-015-0055-5 PMC4542106

[17] Cleophas TJ, Zwinderman AH. *Meta-analysis and Random Effect Analysis*, in *Modern Meta-Analysis: Review and Update of Methodologies*, T.J. Cleophas and A.H. Zwinderman, Editors, Springer International Publishing: Cham. 2017; 51–62.

[18] Xiao M, et al. Controversy and debate: questionable utility of the relative risk in clinical research: paper 4 :odds ratios are Far from portable - A call to use realistic models for effect variation in meta-analysis. J Clin Epidemiol. 2022;142:294–304. 34390790 10.1016/j.jclinepi.2021.08.002 PMC8831641

[19] Cairns M, Prendergast LA. On ratio measures of heterogeneity for meta-analyses. Res Synth Methods. 2022;13(1):28–47. 34328266 10.1002/jrsm.1517

[20] Ioannidis JPA. The importance of predefined rules and prespecified statistical analyses: do not abandon significance. JAMA. 2019;321(21):2067–8. 30946431 10.1001/jama.2019.4582

[21] Huang Y, et al. Comparison between robot- and video-assisted thoracoscopic surgeries for anterior mediastinal lesions. Eur J Cardiothorac Surg. 2025. 10.1093/ejcts/ezaf113. 40323308 10.1093/ejcts/ezaf113

[22] Trabalza Marinucci B, et al. Robotic versus sternotomy, thoracotomy and video-thoracoscopy approaches for thymoma resection: a comparative analysis of short-term results. J Pers Med. 2025. 10.3390/jpm15010034. 39852226 10.3390/jpm15010034 PMC11767127

[23] Şehitogullari A, et al. Comparison of perioperative outcomes of videothoracoscopy and robotic surgical techniques in thymoma. Asian J Surg. 2020;43(1):244–50. 31047770 10.1016/j.asjsur.2019.04.005

[24] El-Akkawi AI, Eckardt J. Comparison of surgical outcomes after robotic assisted thoracic surgery, video-assisted thoracic surgery and open resection of thymoma. Mediastinum. 2021;5:11. 35118317 10.21037/med-20-56 PMC8794456

[25] Imielski B, et al. Comparative effectiveness and cost-efficiency of surgical approaches for thymectomy. Surgery. 2020;168(4):737–42. 32641277 10.1016/j.surg.2020.04.037 PMC7816338

[26] Jiang B, et al. Robot-assisted thymectomy in large anterior mediastinal tumors: A comparative study with video-assisted thymectomy and open surgery. Thorac Cancer. 2023;14(3):267–73. 36433677 10.1111/1759-7714.14744 PMC9870738

[27] Ye B, et al. Video-assisted thoracoscopic surgery versus robotic-assisted thoracoscopic surgery in the surgical treatment of Masaoka stage I thymoma. World J Surg Oncol. 2013;11:157. 23870330 10.1186/1477-7819-11-157 PMC3716986

[28] Chao YK, et al. Robot-assisted surgery outperforms video-assisted thoracoscopic surgery for anterior mediastinal disease: a multi-institutional study. J Robot Surg. 2024;18(1):21. 38217569 10.1007/s11701-023-01745-1

[29] Zheng C, et al. Outcomes of robot-assisted versus video-assisted mediastinal mass resection during the initial learning curve. J Robot Surg. 2024;18(1):81. 38367155 10.1007/s11701-024-01828-7 PMC10874309

[30] Yang CJ, et al. A National analysis of open versus minimally invasive thymectomy for stage I to III thymoma. J Thorac Cardiovasc Surg. 2020;160(2):555–e56715. 32245668 10.1016/j.jtcvs.2019.11.114

[31] Alvarado CE, et al. Robotic approach has improved outcomes for minimally invasive resection of mediastinal tumors. Ann Thorac Surg. 2022;113(6):1853–8. 34217691 10.1016/j.athoracsur.2021.05.090

[32] Salfity HV, et al. Minimally invasive surgery in the management of resectable thymoma: a retrospective analysis from the National Cancer Database. J Thorac Dis. 2021;13(11):6353–62. 34992815 10.21037/jtd-20-2660 PMC8662507

[33] Peng H, et al. Clinical efficiency of three-port inflatable robot-assisted thoracoscopic surgery in mediastinal tumor resection. World J Surg Oncol. 2024;22(1):83. 38523264 10.1186/s12957-024-03357-x PMC10962077

[34] Yang C, et al. Perioperative outcomes comparison of robotic and video-assisted thoracoscopic thymectomy for thymic epithelial tumor: a single-center experience. Updates Surg. 2024;76(4):1511–9. 38060172 10.1007/s13304-023-01702-5

[35] Kamel MK, et al. Robotic thymectomy: learning curve and associated perioperative outcomes. J Laparoendosc Adv Surg Tech A. 2017;27(7):685–90. 28121481 10.1089/lap.2016.0553

[36] Li R, et al. Comparison of perioperative outcomes between robotic-assisted and video-assisted thoracoscopic surgery for mediastinal masses in patients with different body mass index ranges: A population-based study. Front Surg. 2022;9:963335. 35910463 10.3389/fsurg.2022.963335 PMC9329668

[37] Zeng L, et al. Uniportal video-assisted thoracoscopic surgery and robot-assisted thoracoscopic surgery are feasible approaches with potential advantages in minimally invasive mediastinal lesions resection. Gland Surg. 2021;10(1):101–11. 33633967 10.21037/gs-20-536 PMC7882349

[38] Qian L, et al. A comparison of three approaches for the treatment of early-stage thymomas: robot-assisted thoracic surgery, video-assisted thoracic surgery, and median sternotomy. J Thorac Dis. 2017;9(7):1997–2005. 28839999 10.21037/jtd.2017.06.09 PMC5543005

[39] Kamel MK, et al. National trends and perioperative outcomes of robotic resection of thymic tumours in the united states: a propensity matching comparison with open and video-assisted thoracoscopic approaches†. Eur J Cardiothorac Surg. 2019;56(4):762–9. 31321412 10.1093/ejcts/ezz111

[40] Rowse PG, et al. Minimally invasive thymectomy: the Mayo clinic experience. Ann Cardiothorac Surg. 2015;4(6):519–26. 26693147 10.3978/j.issn.2225-319X.2015.07.03 PMC4669247

[41] Zheng Q, et al. Comparison of three methods in the surgical treatment of mediastinal roof tumors. J Thorac Dis. 2024;16(10):6368–80. 39552888 10.21037/jtd-24-946 PMC11565363

[42] Liu Q, et al. Efficacy of the Da Vinci robot versus thoracoscopic surgery for patients with mediastinal tumors of different body mass index: a multicenter propensity score-matched study. World J Surg Oncol. 2024;22(1):257. 39342280 10.1186/s12957-024-03542-y PMC11439245

[43] Ochi T, et al. Robot-assisted thoracic surgery versus video-assisted thoracic surgery for mediastinal lesions. J Thorac Dis. 2023;15(7):3840–8. 37559661 10.21037/jtd-23-377 PMC10407470

[44] Suda T, et al. Thymectomy via a subxiphoid approach: single-port and robot-assisted. J Thorac Dis. 2016;8(Suppl 3):S265–71. 27014473 10.3978/j.issn.2072-1439.2016.02.34 PMC4783725

[45] Li XK, et al. Comparison of the progression-free survival between robot-assisted thymectomy and video-assisted thymectomy for thymic epithelial tumors: a propensity score matching study. J Thorac Dis. 2020;12(8):4033–43. 32944315 10.21037/jtd-20-1065 PMC7475562

[46] Jun Y, et al. Da Vinci robot-assisted system for thymectomy: experience of 55 patients in China. Int J Med Robot. 2014;10(3):294–9. 24573969 10.1002/rcs.1577

[47] Chiba Y, et al. Robot-assisted and video-assisted thoracoscopic surgery for thymoma: comparison of the perioperative outcomes using inverse probability of treatment weighting method. Gland Surg. 2022;11(8):1287–300. 36082085 10.21037/gs-22-333 PMC9445717

[48] Dong Z, et al. Advantages of robot-assisted resection of large mediastinal tumors: a single-center preliminary study. J Robot Surg. 2024;18(1):190. 38693421 10.1007/s11701-024-01958-y

[49] Hong Z, et al. A comparative study of robotic surgery and thoracoscopic surgery for mediastinal cysts. BMC Surg. 2023;23(1):102. 37118795 10.1186/s12893-023-01994-9 PMC10148468

[50] Meacci E, et al. Learning curve of Robot-Assisted thymectomy: single surgeon’s 7-Year experience. Front Surg. 2022;9:860899. 36034391 10.3389/fsurg.2022.860899 PMC9415802

[51] Shen C, et al. Robot-assisted thoracic surgery versus video-assisted thoracic surgery for treatment of patients with thymoma: A systematic review and meta-analysis. Thorac Cancer. 2022;13(2):151–61. 34806328 10.1111/1759-7714.14234 PMC8758429

[52] Dang J, et al. Meta-analysis of clinical efficacy of thoracoscopy and robotic surgery in the treatment of mediastinal tumors. World J Surg Oncol. 2024;22(1):70. 38413953 10.1186/s12957-024-03325-5 PMC10900664

[53] Wu WJ, et al. Does robotic-assisted thymectomy have advantages over video-assisted thymectomy in short-term outcomes? A systematic view and meta-analysis. Interact Cardiovasc Thorac Surg. 2021;33(3):385–94. 33997899 10.1093/icvts/ivab109 PMC8691671

[54] Bongiolatti S, et al. Long-term outcomes of robot-assisted radical thymectomy for large thymomas: A propensity matched analysis. Int J Med Robot. 2022;18(5):e2439. 35830541 10.1002/rcs.2439

[55] Huang L, et al. Robotic-assisted extended thymectomy for large resectable thymoma: 21 years’ experience. J Thorac Cardiovasc Surg. 2025;169(2):469–e48310. 39159885 10.1016/j.jtcvs.2024.08.005

[56] Falkson CB, et al. Surgical, Radiation, and systemic treatments of patients with thymic epithelial tumors: A systematic review. J Thorac Oncol. 2023;18(3):299–312. 36343922 10.1016/j.jtho.2022.10.016

