# Supplementary material for: Comparative meta-analysis of robot- and video-assisted surgery for thymoma: efficacy, learning curve, and economic burden in 7347 patients
Source: World J Surg Oncol. 2025 Nov 29;24:14. doi: 10.1186/s12957-025-04132-2 (PMC12771877; doi:10.1186/s12957-025-04132-2)
Supplement: Supplementary file 1 — Supplementary Material 1 [file 12957_2025_4132_MOESM1_ESM.docx]

| **Section and Topic** | **Item #** | **Checklist item** | **Location where item is reported** |
| --- | --- | --- | --- |
| **TITLE** | | |  |
| Title | 1 | Comparative Meta-analysis of Robot- and Video-assisted Surgery for Thymoma: Efficacy, Learning Curve, and Economic Burden in 7347 Patients |  |
| **ABSTRACT** | | |  |
| Abstract | 2 | Objective: This meta-analysis systematically evaluates the perioperative outcomes, learning curve, and hospitalization costs of robot-assisted thoracoscopic surgery (RATs) compared to video-assisted thoracoscopic surgery (VATs) for thymoma resection.  Methods: A comprehensive literature search was conducted in PubMed, Embase, Web of Science, and the Cochrane Library, with the final update in June 2025, following PRISMA guidelines. Constant-effects model or random-effects model was used based on heterogeneity, and subgroup analyses were performed accordingly.  Results: Seventeen studies with a total of 7,347 patients (RATs: 3,122; VATs: 4,225) were included. Compared to VATs, RATs had significantly lower rates of conversion to open surgery, shorter operative time, less intraoperative blood loss, lower chest tube drainage volume and duration, higher rates of R0 resection, shorter postoperative hospital stays, and fewer complications, including pulmonary infections. Analysis of the learning curve in 153 RATs patients showed operative times significantly decreased after 20 procedures. Total hospitalization costs were higher for RATs, there were no significant differences in 30-day or 90-day mortality between groups.  Conclusion: RATs is associated with superior perioperative outcomes, including faster recovery and fewer complications, suggesting distinct clinical advantages in thymoma surgery. Despite higher hospitalization costs, the benefits in clinical efficacy support the broader adoption of RAT. Large-scale, prospective studies are warranted to further validate these findings. |  |
| **INTRODUCTION** | | |  |
| Rationale | 3 | **Synthesize fragmented evidence** from multiple studies to provide pooled estimates of survival, recurrence, and treatment efficacy.  **Clarify the role of adjuvant therapy** across different disease stages and histologic subtypes.  **Identify robust prognostic factors** that can better stratify patient risk and inform treatment planning.  **Update clinical practice** with the most recent and highest-quality data, supporting guidelines and decision-making in the management of this rare tumor. |  |
| Objectives | 4 | This meta-analysis systematically evaluates the perioperative outcomes, learning curve, and hospitalization costs of robot-assisted thoracoscopic surgery (RATs) compared to video-assisted thoracoscopic surgery (VATs) for thymoma resection. |  |
| **METHODS** | | |  |
| Eligibility criteria | 5 | A total of 30 studies were included in the analysis, involving 7,347 patients. Of these, 3,122 patients underwent RATs, and 4,225 patients received VATs. |  |
| Information sources | 6 | We conducted systematic searches in PubMed/MEDLINE, Embase (Embase.com), the Cochrane Library (CENTRAL), and the Web of Science Core Collection, and supplemented the searches with trial registration platforms such as ClinicalTrials.gov, WHO ICTRP, EU CTR, ISRCTN, ChiCTR, ANZCTR, and UMIN-CTR; we reviewed gray literature sources including medRxiv, Research Square, ProQuest Dissertations & Theses Global, and BASE; we visited guideline/society websites such as NCCN, ESMO, ITMIG, and NCI PDQ; and we performed backward citation tracking and forward citation tracking (via Web of Science and Google Scholar) on the included studies and references from related reviews/guidelines. All sources were searched from their inception up to June 30, 2025. |  |
| Search strategy | 7 | We conducted a systematic review and meta-analysis following PRISMA guidelines, with the most recent literature update on June 30, 2025. The literature was retrieved from major databases including PubMed, Embase, Cochrane Library, and Web of Science. In PubMed, the search strategy used was: “((((thymoma) OR (Thymoma)) OR (mediastinal tumor)) OR (mediastinal)) AND ((((((da Vinci) OR (Robot)) OR (robot)) OR (robotic)) OR (robot-assisted) OR (robotic-assisted) OR (robotic-assisted thoracic surgery)) AND (((((((vat) OR (VATs)) OR (VAT)) OR (Thoracoscope)) OR (video assisted thoracic surgery)) OR (video)) OR (thoracoscopic)))”. In Embase, the search strategy was: “‘thymoma’/exp OR thymoma OR ‘mediastinal tumor’/exp OR ‘mediastinal tumor’ AND (‘da Vinci’:ti,ab OR ‘robot*’:ti,ab OR ‘robot assisted’:ti,ab OR ‘robotic thoracic surgery’:ti,ab) AND (‘vat*’:ti,ab OR ‘video assisted thoracoscopic surgery’:ti,ab OR thoracoscop*:ti,ab OR ‘video surgery’:ti,ab)”. For Cochrane Library (CENTRAL), the search strategy was: “(thymoma OR mediastinal tumor) AND (robot OR robotic OR ‘da Vinci’ OR ‘robot-assisted’ OR ‘robotic-assisted thoracic surgery’) AND (VAT OR VATs OR ‘video assisted thoracoscopic surgery’ OR thoracoscopic OR video)”. In Web of Science, the search strategy used was: “TS=(thymoma OR ‘mediastinal tumor’) AND TS=(robot OR robotic OR ‘robot-assisted’ OR ‘da Vinci’) AND TS=(‘VAT’ OR ‘VATs’ OR ‘video-assisted thoracoscopic surgery’ OR thoracoscopic OR video)”. For clinical trial registers, searches were conducted on ClinicalTrials.gov and WHO ICTRP, with the keyword: “thymoma AND (robot OR robotic OR da Vinci)”. Additionally, grey literature and preprints were sourced from medRxiv and Research Square, using the search terms “thymoma AND robotic” or “thymoma AND video-assisted”. ProQuest Dissertations & Theses Global was searched using “thymoma AND (robot* OR video-assisted)”. We also reviewed guidelines and resources from NCCN, ESMO, ITMIG, and NCI PDQ. Lastly, reference lists from all included studies and relevant systematic reviews were manually screened, and forward citation tracking was performed using Web of Science and Google Scholar’s “Cited by” function to ensure all relevant literature was captured. The latest search date for all sources was June 30, 2025. |  |
| Selection process | 8 | **Study Selection Methodology**  In this systematic review and meta-analysis, the determination of whether a study met the inclusion criteria followed the PRISMA guidelines. The screening process was carried out by two independent reviewers (Reviewer 1 and Reviewer 2) to ensure the quality and impartiality of the process. Each reviewer assessed the records based on the **PICOS framework** (Population, Intervention, Comparison, Outcomes, Study design).   1. **Initial Screening**: Each reviewer screened all the records retrieved from the searches to identify those that met the inclusion criteria. Specifically, the reviewers first conducted a title and abstract screening to exclude studies that were clearly unrelated to the research question or did not meet the inclusion criteria. A total of **602 records** were initially screened. Each reviewer independently completed this step, ensuring a comprehensive and unbiased process. 2. **Full-Text Screening**: For the records that passed the initial screening, the reviewers then reviewed the full texts to determine whether they met the final inclusion criteria. This step involved a more detailed examination, particularly focusing on study design, intervention measures, outcome indicators, and the inclusion of the study population. In the end, **30 studies** were included in the analysis, involving **7,347 patients**. Of these, 3,122 patients underwent robot-assisted thymectomy (RAT), and 4,225 patients underwent video-assisted thymectomy (VAT). 3. **Independence and Collaboration**: All screening tasks were performed independently by the two reviewers to ensure fairness in the selection process. In cases of disagreements, the reviewers discussed the issue and reached a consensus, with a third reviewer (Reviewer 3) involved to resolve any disputes if necessary. 4. **Use of Automated Tools**: During the screening process, we utilized automated tools such as **EndNote** and **Rayyan** (a collaborative screening platform) to manage the reference library and minimize duplicate records. The Rayyan tool assisted with artificial intelligence-assisted screening, enabling faster identification of studies likely to meet the inclusion criteria, as well as automatic de-duplication and tracking of screening decisions. However, the final decision on inclusion was made independently by the reviewers. 5. **Transparency in the Screening Process**: We ensured that the screening process was transparent and that each decision was traceable. All screening decisions were reviewed by the reviewers and a third-party reviewer to minimize potential bias. |  |
| Data collection process | 9 | **Data Collection Methods**  Data extraction for this systematic review and meta-analysis followed a structured and standardized process to ensure consistency and accuracy. The data collection process was conducted by two independent reviewers (Reviewer 1 and Reviewer 2), who worked in parallel to ensure the reliability of the results.   1. **Data Points Collected**: Each reviewer independently extracted data from the selected studies. The data collected included the following key variables:    - Study characteristics: publication year, surgical category, sample size, patient demographics (age, sex, BMI), tumor size, WHO histological classification, surgical approach, myasthenia gravis (MG) status.    - Perioperative outcomes: conversion rate to open surgery, operative time, intraoperative blood loss, drainage volume and duration, R0 resection rate, postoperative length of stay, complication rates (including pulmonary infection), total hospitalization costs, learning curve metrics, and 30- and 90-day mortality.   Each reviewer extracted the relevant data from each study independently. The total number of data points collected from each study varied depending on the outcomes reported and the completeness of the data.   1. **Independent Work**: The data extraction process was carried out independently by the two reviewers to minimize bias. After extracting the data, any discrepancies between the two reviewers' results were resolved through discussion, and when necessary, a third reviewer (Reviewer 3) was consulted to reach a consensus. 2. **Confirmation of Data from Investigators**: In cases where the data was incomplete, unclear, or inconsistent between studies, the corresponding authors were contacted to request clarification or additional data. This step ensured that the data used in the analysis was accurate and comprehensive. Contacting study authors was particularly relevant for variables such as detailed complication rates, missing outcome measures, or unclear definitions of specific perioperative events. 3. **Use of Automated Tools**: Automated tools were used to assist in managing and organizing the data collection process. We employed **EndNote** for reference management and **Rayyan** for initial screening of studies. For data extraction, we used **Covidence**, a tool that allows for collaborative data extraction. Covidence helped streamline the process by providing a platform for the reviewers to enter data, track discrepancies, and resolve conflicts. The use of Covidence also enabled automatic comparison of the data extracted by both reviewers and flagged inconsistencies for review. 4. **Data Verification and Final Confirmation**: Once data extraction was completed, a final verification step was carried out. The data was double-checked by both reviewers to ensure consistency and accuracy. If discrepancies arose during this step, the relevant study was revisited, and the data was reviewed with input from a third reviewer when necessary. All extracted data was stored in a secure, shared database to facilitate easy access and future reference.   . |  |
| Data items | 10a | **Outcomes Sought for Data Collection**  The following outcomes were defined prior to the systematic review and meta-analysis, and data were sought for each of these outcome domains. We aimed to collect data on all measures, time points, and analyses related to each outcome, but in cases where not all results were compatible or available, specific criteria were used to determine which results to collect.  **1. Conversion Rate to Open Surgery**   - **Definition**: The proportion of patients who required conversion from minimally invasive surgery (robot-assisted or video-assisted thoracoscopic surgery) to open surgery during the procedure. - **Data Collected**: We sought data on the conversion rates from all included studies, regardless of the time points or subgroup variations (e.g., based on tumor size or surgeon experience). - **Criteria for Collection**: If studies reported conversion rates at different time points, we prioritized the overall conversion rate for consistency across studies. Only studies with clear and comparable definitions of conversion were included.   **2. Operative Time**   - **Definition**: The total time spent performing the surgical procedure, from the start of the incision to the completion of the procedure. - **Data Collected**: All studies that reported operative time, regardless of the specific surgical approach or patient characteristics, were included. - **Criteria for Collection**: If multiple operative time measurements were reported (e.g., by surgeon or in different phases of the study), the time data representing the overall average or a specific phase (such as the first set of surgeries vs later ones) were included based on consistency.   **3. Intraoperative Blood Loss**   - **Definition**: The total volume of blood lost during surgery, typically measured in milliliters (mL). - **Data Collected**: Data were sought on total intraoperative blood loss for each study, ensuring consistency in measurement units. - **Criteria for Collection**: If blood loss was reported at multiple time points (e.g., during surgery vs post-surgery), the data that represented the primary intraoperative blood loss were prioritized.   **4. Drainage Volume and Duration**   - **Definition**: The amount of fluid drained from the surgical site postoperatively, and the length of time drainage was required. - **Data Collected**: Studies that reported both the volume of drainage (in mL) and the duration of drainage (in days) were included. - **Criteria for Collection**: Only data that explicitly defined both volume and duration were considered. If studies reported multiple types of drains or time points, the data most consistent with the primary outcome domain were collected (i.e., total drainage volume and total duration).   **5. R0 Resection Rate**   - **Definition**: The proportion of patients who underwent complete resection of the tumor with no residual microscopic disease at the resection margin. - **Data Collected**: R0 resection rate data were sought from all included studies, regardless of the specific histological classification or tumor staging. - **Criteria for Collection**: In studies where multiple definitions of R0 resection were used, we focused on those that were most universally applicable across studies (e.g., clear margin definitions).   **6. Postoperative Length of Stay**   - **Definition**: The number of days a patient remains in the hospital following the surgical procedure. - **Data Collected**: Data on the total postoperative length of stay were collected from each study. - **Criteria for Collection**: If studies provided data on length of stay at multiple time points (e.g., day 1 vs day 7 post-surgery), the overall average length of stay was used for consistency across studies.   **7. Complication Rates (Including Pulmonary Infection)**   - **Definition**: The incidence of complications following surgery, with a particular focus on pulmonary infections. - **Data Collected**: Data were sought for all complications reported by each study, but special emphasis was placed on pulmonary infections, given their clinical significance. - **Criteria for Collection**: If studies reported multiple complications (e.g., arrhythmias, wound infections, nerve injuries), the data most relevant to the scope of the review (e.g., respiratory complications) were prioritized.   **8. Total Hospitalization Costs**   - **Definition**: The total cost incurred by a patient during the hospital stay, including surgery, medications, and post-operative care. - **Data Collected**: Studies reporting hospitalization costs were included, particularly those that provided detailed cost breakdowns. - **Criteria for Collection**: If studies reported costs in varying currencies or timeframes, we standardized costs to the same currency and adjusted for inflation where possible, based on the year of publication.   **9. Learning Curve Metrics**   - **Definition**: The relationship between the number of surgeries performed by a surgeon and the improvement in surgical performance (e.g., reduced operative time, fewer complications). - **Data Collected**: Data related to the learning curve were sought, especially studies that provided clear metrics such as changes in operative time or complication rates over time. - **Criteria for Collection**: Only studies that provided clear, longitudinal data showing the learning curve over a series of surgeries were included.   **10. 30-Day and 90-Day Mortality**   - **Definition**: The percentage of patients who died within 30 days or 90 days post-surgery. - **Data Collected**: Data were sought on both 30-day and 90-day mortality rates, where available. - **Criteria for Collection**: If mortality data were reported at different time points, the 30-day and 90-day mortality rates were prioritized. In cases where mortality was reported over a different period, we used the most comparable time frame.   **Data Collection Decisions**  Not all studies reported every outcome for each of the domains above. In such cases, we employed the following methods to decide which results to collect:   - **Prioritization of Primary Data**: When multiple outcomes were reported at different time points or in different subgroups, the most comprehensive and consistent data across studies were prioritized (e.g., overall complication rates vs subgroup analyses). - **Standardization**: Where necessary, we standardized measures (such as currency for hospitalization costs or units for blood loss) to ensure comparability. - **Author Clarification**: In instances where data were incomplete or unclear, corresponding authors were contacted to confirm missing information or clarify ambiguities. Only data confirmed by the authors were included. |  |
|  | 10b | **Other Variables Sought for Data Collection**  In addition to the primary outcomes, data were also collected for several other variables that could provide additional insights into the studies and help contextualize the findings. These variables included participant characteristics, intervention details, and study-specific factors. Below is a list and definition of these variables:  **1. Participant Characteristics**   - **Definition**: Key demographic and clinical characteristics of the study participants, including:   - **Age**: The mean or median age of participants, or age ranges (e.g., <60 years, ≥60 years).   - **Sex**: The number or percentage of male and female participants.   - **Body Mass Index (BMI)**: The mean or median BMI, or the proportion of participants within various BMI categories (e.g., normal weight, overweight, obese).   - **Tumor Size**: The average or median size of the tumor at diagnosis, typically measured in centimeters (cm).   - **Histological Classification (WHO Classification)**: The histological subtype of thymoma, categorized based on the World Health Organization (WHO) classification (e.g., Type A, AB, B1, B2, B3, thymic carcinoma).   - **Myasthenia Gravis (MG) Status**: The proportion of patients with or without myasthenia gravis, a common comorbidity in thymoma patients. - **Data Collected**: The aim was to collect data on these characteristics for all included studies to help assess the generalizability of findings. - **Assumptions about Missing or Unclear Data**:   - When specific demographic data were not provided, we made assumptions based on the overall study population description (e.g., if the study only included older patients, we assumed the mean age was consistent with that population).   - If studies reported categorical data but lacked exact means or medians (e.g., age ranges but no averages), we used available summary statistics or estimated averages from ranges when necessary.   **2. Intervention Characteristics**   - **Definition**: Detailed information about the interventions being studied, including:   - **Type of Surgery**: The type of surgery used in the study (e.g., robot-assisted thymectomy [RAT], video-assisted thoracoscopic surgery [VAT], open surgery).   - **Surgical Approach**: Specific approach used for surgery (e.g., subxiphoid, lateral, or median sternotomy).   - **Surgeon Experience**: The experience level of the surgeon(s) performing the surgery, which may be categorized as novice, intermediate, or expert based on the number of cases performed.   - **Adjuvant Therapy**: Whether patients received any adjuvant treatments (e.g., chemotherapy, radiotherapy) as part of the management, and if so, the specifics of such treatments. - **Data Collected**: Data were sought on the surgical type, approach, and any relevant adjuvant treatments used in each study. Information on surgeon experience, when available, was also collected. - **Assumptions about Missing or Unclear Data**:   - When the exact surgical approach was not detailed, we used information from study descriptions (e.g., if the study was described as minimally invasive, we assumed VAT or RAT was used).   - In studies where adjuvant therapy data were incomplete, we included data only for patients who had not received adjuvant therapy unless it was explicitly stated that adjuvant therapy was used universally.   **3. Study Design and Methodology**   - **Definition**: Characteristics related to how the study was conducted, including:   - **Study Design**: Whether the study was randomized, cohort-based, or retrospective. This also includes details on sample size calculations and randomization procedures when applicable.   - **Follow-up Duration**: The length of time over which participants were followed up and outcomes were measured (e.g., 30 days, 90 days, 1 year).   - **Inclusion and Exclusion Criteria**: The criteria used to select participants for inclusion in the study, and reasons for exclusion of certain individuals. - **Data Collected**: Data were sought on study design, inclusion/exclusion criteria, and follow-up duration to assess the potential for bias and the strength of the evidence. - **Assumptions about Missing or Unclear Data**:   - If inclusion or exclusion criteria were unclear, we assumed standard criteria (e.g., excluding patients with distant metastasis or other major comorbidities) unless otherwise specified in the study.   - When follow-up duration was not clearly reported, we used the shortest follow-up period as the conservative estimate for outcomes.   **4. Funding Sources and Potential Conflicts of Interest**   - **Definition**: Information on the source of funding for the study and any potential conflicts of interest.   - **Funding Source**: Whether the study was funded by government agencies, private industry, or other sources.   - **Conflict of Interest**: Whether the authors declared any financial or professional conflicts of interest related to the study. - **Data Collected**: Funding sources and conflicts of interest declarations were sought to assess the potential for bias in the study design or reporting of outcomes. - **Assumptions about Missing or Unclear Data**:   - If no funding source or conflict of interest statement was provided, we assumed that the study did not report a conflict, unless there were clear indications otherwise in the manuscript.   **5. Statistical Methods and Analysis**   - **Definition**: The statistical methods used to analyze data and report results, including:   - **Statistical Models**: Whether the studies used fixed-effects or random-effects models for their analyses.   - **Sensitivity Analyses**: Whether sensitivity analyses were performed to test the robustness of the findings.   - **Handling of Missing Data**: How missing data were handled in the study (e.g., imputation methods, exclusion of incomplete cases). - **Data Collected**: Data on statistical methods used in the analysis, including sensitivity analyses and handling of missing data, were collected to assess the quality and reliability of the reported results. - **Assumptions about Missing or Unclear Data**:   - If the statistical method was unclear, we assumed the most commonly used method (e.g., random-effects models for meta-analysis) and reported any deviations.   - Missing data handling was assumed to follow standard methods (e.g., exclusion of missing data for complete case analysis) unless otherwise reported.   **Assumptions Made About Missing or Unclear Information**  In instances where data were missing or unclear, the following assumptions were made:   - If participant or intervention characteristics were reported in broad categories (e.g., age range, surgical approach) but not as specific values, we estimated averages or used the midpoint of ranges when possible. - For studies that reported incomplete outcome data (e.g., only median values without measures of variability), we used standard methods for converting medians to means (e.g., the Hozo method for continuous data) and reported ranges where appropriate. - When studies did not report exact statistical details (e.g., exact p-values), we used available summary data (e.g., confidence intervals, odds ratios) and considered results as indicative rather than definitive. |  |
| Study risk of bias assessment | 11 | **Risk of Bias Assessment**  To assess the risk of bias in the included studies, we followed established guidelines and employed well-recognized tools for evaluating methodological quality and potential biases in the studies. Below are the methods used for this process:  **1. Tools Used for Risk of Bias Assessment**   - **Newcastle-Ottawa Scale (NOS)**: For cohort studies, we used the **Newcastle-Ottawa Scale (NOS)**, which is widely used to assess the risk of bias in observational studies. This tool evaluates three broad areas of study design:   - **Selection**: The process by which participants were selected.   - **Comparability**: Whether groups were comparable based on relevant factors.   - **Exposure/Outcome**: The outcome of interest and whether it was measured appropriately. The NOS uses a star-based system (maximum of 9 stars) to rate studies, with 6 or more stars indicating an acceptable quality and 8-9 stars indicating high quality. - **Cochrane Risk of Bias Tool**: For randomized controlled trials (RCTs), we applied the **Cochrane Risk of Bias tool**, which provides a comprehensive evaluation of six domains:   - **Random sequence generation** (selection bias).   - **Allocation concealment** (selection bias).   - **Blinding** (performance and detection biases).   - **Incomplete outcome data** (attrition bias).   - **Selective reporting** (reporting bias).   - **Other sources of bias** (such as early stopping or funding bias). The Cochrane tool provides a judgment of low, high, or unclear risk for each domain. - **ROBINS-I Tool (Risk Of Bias In Non-randomized Studies – of Interventions)**: In cases where the studies were non-randomized intervention studies (e.g., cohort studies with surgical interventions), we applied the **ROBINS-I tool**, which assesses the risk of bias across seven domains:   - **Confounding**.   - **Participant selection**.   - **Classification of interventions**.   - **Deviation from intended interventions**.   - **Missing data**.   - **Measurement of outcomes**.   - **Selection of the reported result**.   **2. Reviewers and Independence**   - **Number of Reviewers**: Two independent reviewers (Reviewer 1 and Reviewer 2) assessed the risk of bias for each included study. Both reviewers independently completed the risk of bias assessments for each study. - **Independence**: The two reviewers worked independently to ensure impartiality in their assessments. If there were discrepancies between their assessments, they discussed the differences and resolved the issues through consensus. In case consensus could not be reached, a third reviewer (Reviewer 3) was involved to make the final decision.   **3. Use of Automation Tools**   - **EndNote and Rayyan**: While no specific automation tool was used for the actual risk of bias assessment, we utilized **Rayyan** for initial study screening and **EndNote** for reference management. These tools helped manage the large volume of studies and ensured efficient organization and removal of duplicate records during the selection process. - **Manual Risk of Bias Assessment**: The actual risk of bias assessments were carried out manually by the reviewers using the aforementioned tools (NOS, Cochrane Risk of Bias, and ROBINS-I). This process was carried out without the use of automated tools to ensure accuracy and clarity in the assessment of each bias domain.   **4. Assessment of Discrepancies and Consensus**   - Discrepancies between the reviewers were handled through direct discussion, and when necessary, the involvement of a third reviewer to resolve conflicts. The final risk of bias judgment was based on consensus between the two reviewers, ensuring that all relevant factors were considered.   **5. Judgment of Risk of Bias**   - **Low Risk**: Studies with no or minimal bias, where the study design and execution were unlikely to affect the results. - **High Risk**: Studies with major flaws in design, execution, or reporting that could lead to significant bias and affect the validity of the findings. - **Unclear Risk**: Studies where insufficient information was available to make a clear judgment regarding bias. |  |
| Effect measures | 12 | **Effect Measures for Each Outcome**  For each of the outcomes assessed in this systematic review and meta-analysis, specific effect measures were used to synthesize and present the results. These effect measures were chosen based on the nature of the outcome data (e.g., dichotomous vs continuous) and the goal of the analysis.  **1. Conversion Rate to Open Surgery**   - **Effect Measure**: **Odds Ratio (OR)** - **Description**: The odds ratio was used to compare the odds of conversion to open surgery between the robot-assisted thoracoscopic surgery (RAT) group and the video-assisted thoracoscopic surgery (VAT) group. - **Reason for Choice**: As this is a dichotomous outcome (conversion vs no conversion), the odds ratio is an appropriate measure to summarize the relative likelihood of conversion between the two groups.   **2. Operative Time**   - **Effect Measure**: **Mean Difference (MD)** - **Description**: The mean difference was used to compare the average operative time between RAT and VAT groups. - **Reason for Choice**: Operative time is a continuous outcome, and the mean difference is appropriate for comparing the average differences between two groups.   **3. Intraoperative Blood Loss**   - **Effect Measure**: **Mean Difference (MD)** - **Description**: The mean difference was used to compare the average intraoperative blood loss between the RAT and VAT groups. - **Reason for Choice**: As this outcome is measured on a continuous scale (mL), the mean difference is used to compare the average blood loss between the two surgical approaches.   **4. Drainage Volume and Duration**   - **Effect Measure**: **Mean Difference (MD)** - **Description**: The mean difference was used to compare both the total drainage volume (in mL) and the duration of drainage (in days) between RAT and VAT groups. - **Reason for Choice**: Both drainage volume and duration are continuous variables, making the mean difference the appropriate measure to summarize the differences between the two groups.   **5. R0 Resection Rate**   - **Effect Measure**: **Odds Ratio (OR)** - **Description**: The odds ratio was used to compare the odds of achieving an R0 resection (complete resection with clear margins) between the RAT and VAT groups. - **Reason for Choice**: R0 resection is a dichotomous outcome (R0 resection vs non-R0 resection), so the odds ratio is appropriate to assess the relative likelihood of achieving complete resection between the two groups.   **6. Postoperative Length of Stay**   - **Effect Measure**: **Mean Difference (MD)** - **Description**: The mean difference was used to compare the average length of stay in the hospital following surgery between the RAT and VAT groups. - **Reason for Choice**: Postoperative length of stay is a continuous variable, so the mean difference is the appropriate measure to compare the average hospital stay between the two surgical approaches.   **7. Complication Rates (Including Pulmonary Infection)**   - **Effect Measure**: **Odds Ratio (OR)** - **Description**: The odds ratio was used to compare the odds of experiencing any complications (including pulmonary infections) between the RAT and VAT groups. - **Reason for Choice**: As complication rates are dichotomous (complication vs no complication), the odds ratio is the appropriate effect measure to compare the likelihood of complications between the two groups.   **8. Total Hospitalization Costs**   - **Effect Measure**: **Mean Difference (MD)** - **Description**: The mean difference was used to compare the total hospitalization costs between RAT and VAT groups. - **Reason for Choice**: Hospitalization costs are typically measured as continuous data, so the mean difference was used to compare the average cost differences between the two surgical approaches.   **9. Learning Curve Metrics**   - **Effect Measure**: **Mean Difference (MD)** - **Description**: The mean difference was used to compare the operative time or complication rates during the learning curve phase of RAT versus VAT procedures. - **Reason for Choice**: As the learning curve metrics (such as operative time) are continuous, the mean difference is the most appropriate effect measure for comparing the differences in outcomes over time between the two techniques.   **10. 30-Day and 90-Day Mortality**   - **Effect Measure**: **Odds Ratio (OR)** - **Description**: The odds ratio was used to compare the odds of mortality at 30 days and 90 days between the RAT and VAT groups. - **Reason for Choice**: Mortality is a dichotomous outcome (alive vs deceased), so the odds ratio is used to measure the relative odds of death between the two groups at each time point. |  |
| Synthesis methods | 13a | **Processes for Deciding Study Eligibility for Each Synthesis**  The process of deciding which studies were eligible for inclusion in each synthesis followed a clear and systematic approach. This approach involved careful screening and categorization of studies based on predefined inclusion criteria, intervention characteristics, and outcome measures. Below is a detailed description of the steps taken:  **1. Defining Inclusion Criteria**   - **Predefined Eligibility Criteria**: Prior to starting the study selection process, eligibility criteria were clearly defined using the **PICOS framework** (Population, Intervention, Comparison, Outcome, Study design). Only studies that met all of these criteria were considered for inclusion in the synthesis.   - **Population**: Studies involving patients undergoing thymectomy for thymoma.   - **Intervention**: Robot-assisted thymectomy (RAT) or video-assisted thoracoscopic thymectomy (VAT).   - **Comparison**: Studies comparing RAT to VAT.   - **Outcome**: Relevant perioperative outcomes, complications, costs, and survival data.   - **Study Design**: Randomized controlled trials (RCTs), cohort studies, and other comparative studies. - **Exclusion Criteria**: Studies were excluded if they were:   - Case reports, reviews, conference abstracts, or single-arm studies.   - Studies published in languages other than English.   - Studies that did not provide sufficient or reliable data on the required outcomes.   **2. Tabulating Intervention Characteristics**   - **Study Intervention Classification**: To ensure that only studies comparing **robot-assisted thymectomy (RAT)** and **video-assisted thoracoscopic thymectomy (VAT)** were included, the intervention characteristics were tabulated and compared against the predefined intervention groups.   - Studies were categorized based on the specific surgical approach used (RAT vs VAT).   - If studies reported mixed interventions (e.g., some patients receiving RAT and others receiving VAT), they were included only if data for each group were reported separately or if subgroup data could be extracted.   **3. Comparing Against Planned Groups**   - **Defining Groups for Synthesis**: Based on the inclusion and exclusion criteria, we categorized the studies into planned groups for each synthesis:   - **Group 1**: Robot-assisted thymectomy (RAT)   - **Group 2**: Video-assisted thoracoscopic thymectomy (VAT)   - **Group 3**: Comparison group (if studies included both RAT and VAT for comparison, we grouped them accordingly). - **Eligibility for Each Synthesis**: Each study was reviewed to ensure it provided data specific to the interventions of interest (RAT vs VAT). Studies that did not report direct comparisons between RAT and VAT, or studies that lacked data on the planned outcomes (e.g., complication rates, operative time), were excluded from the synthesis.   **4. Assessment of Study Designs**   - **Study Design Compatibility**: Studies were assessed for their design compatibility with the synthesis. RCTs and cohort studies were prioritized for inclusion, as they provided the most robust evidence for comparing RAT and VAT. If a study used a different design, it was included only if it reported outcomes that were comparable across the two groups (RAT vs VAT). - **Data Availability and Completeness**: Only studies that reported the required outcomes were included in the synthesis. If a study did not report outcomes that were necessary for the meta-analysis (e.g., operative time, conversion rates), it was excluded from the synthesis of that specific outcome.   **5. Use of Subgroup Analyses**   - **Subgroup Definitions**: If studies reported additional factors that might influence the outcomes (e.g., tumor size, surgeon experience, approach used), subgroup analyses were planned to assess whether these factors influenced the comparative effectiveness of RAT vs VAT.   - For example, if a study presented results for RAT vs VAT in both early-stage and advanced-stage thymomas, we planned to perform a subgroup analysis for each stage of disease to evaluate any differences in outcomes. - **Eligibility Based on Subgroups**: Studies that provided data on relevant subgroups (e.g., age, sex, tumor size, approach) were eligible for inclusion in subgroup analyses. These analyses were aimed at exploring how different factors might influence the outcomes of interest.   **6. Handling Missing or Incomplete Data**   - **Data Imputation**: If a study provided incomplete data or lacked certain outcome measures, we attempted to contact the study authors to request the missing data. If the missing data could not be obtained, the study was excluded from the synthesis for that particular outcome.   - In cases where studies reported summary statistics (e.g., medians, interquartile ranges), we attempted to convert these to means and standard deviations using standard methods, as outlined in the data collection section. - **Deciding Which Results to Collect**: In instances where multiple outcome measures were reported (e.g., multiple time points for postoperative complications), we selected the most consistent and relevant results for each outcome domain. The decision was based on factors such as the most commonly reported outcome measures across studies, as well as the statistical methodology used in the analysis.   **7. Final Inclusion Decisions**   - After applying the predefined inclusion and exclusion criteria, and ensuring the study characteristics matched the planned intervention groups, the studies that met all criteria were included in the final synthesis. The final selection was made based on the clarity of the reported data and the study's relevance to the research question. |  |
|  | 13b | **Methods for Preparing Data for Presentation or Synthesis**  To ensure the accurate synthesis and presentation of results, several methods were employed to prepare the data from the included studies. These methods addressed challenges such as missing summary statistics, data conversions, and ensuring consistency across studies. Below is a detailed description of the steps taken:  **1. Handling Missing Data**   - **Contacting Study Authors**: If a study did not report key outcome measures (e.g., means, standard deviations, or sample sizes), the corresponding author was contacted to request the missing data. If the data could not be obtained within the study’s timeline, the study was excluded from the synthesis for that specific outcome. - **Exclusion of Studies with Incomplete Data**: Studies that did not provide sufficient data (e.g., no reported outcomes or incomplete reporting) were excluded from the synthesis for the corresponding outcome. Incomplete data were defined as instances where the outcome of interest could not be derived or calculated based on the provided summary statistics.   **2. Handling Missing Summary Statistics**   - **Imputation of Missing Values**: In cases where a study reported medians and interquartile ranges (IQR) but not means and standard deviations (SD), we used the **Hozo method** to estimate means and SDs based on the provided median and IQR. This method is commonly used when only non-parametric summary statistics are reported and helps in converting data to a form compatible with meta-analysis.   - Formula: , (based on an assumed normal distribution).  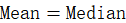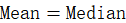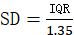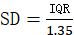  - **For Proportions**: If proportions were missing (e.g., for conversion rates or complication rates), we calculated them from raw data when available (e.g., number of events over the total number of patients). If this information was not reported, and no other summary statistics were provided, the study was excluded for that specific outcome.   **3. Data Conversions for Consistency**   - **Standardizing Units**: For outcomes like blood loss, drainage volume, and hospitalization costs, different studies used different units (e.g., milliliters vs. liters, or different currencies). All units were converted to the same scale to ensure comparability. For blood loss and drainage volume, conversions were made to milliliters (mL) as the standard unit.   - For costs, all values were converted to **USD** using the exchange rate at the time of publication, adjusting for inflation where applicable. - **Handling Multiple Time Points**: Some studies reported outcomes at multiple time points (e.g., postoperative complications at 30 days, 90 days, and 1 year). In these cases:   - **Preferred Time Point**: The time point that was most commonly reported across studies (e.g., 30-day mortality) was prioritized for inclusion.   - If multiple time points were relevant (e.g., 30-day and 90-day mortality), the data for each time point were kept separate and synthesized accordingly.   **4. Standardizing Outcome Measures**   - **Consistency in Definitions**: To ensure comparability of results, we standardized the definitions of key outcomes across studies. For example, “conversion to open surgery” was uniformly defined as any patient who required a transition from a minimally invasive approach (RAT or VAT) to an open surgical procedure. Any study that used a different definition was excluded from the synthesis for that outcome. - **Surgical Approach Classification**: If studies reported different types of minimally invasive surgery (e.g., RAT vs. VAT), the study was included only if clear data on both surgical approaches were provided. Studies that used mixed surgical approaches (e.g., some patients in the study received RAT while others received VAT) were included only if subgroup data for each group were separately reported.   **5. Data Aggregation for Meta-Analysis**   - **Pooling Data from Different Studies**: Where possible, we pooled the data from studies that reported similar outcomes. For continuous outcomes (e.g., operative time, blood loss, length of stay), the mean difference (MD) was used to combine the results from different studies. For dichotomous outcomes (e.g., conversion rates, complication rates), the odds ratio (OR) was used.   - If studies used different statistical models (fixed-effects vs. random-effects), the data were aggregated using random-effects models to account for variability between studies.   **6. Assessing and Handling Study Heterogeneity**   - **I² Statistic**: The **I² statistic** was calculated to assess the degree of heterogeneity among the studies. This statistic indicates the percentage of total variation across studies that is due to heterogeneity rather than chance. A value of 25%, 50%, or 75% was considered low, moderate, or high heterogeneity, respectively. - **Sensitivity Analyses**: To test the robustness of the synthesis, sensitivity analyses were performed to explore whether specific studies or data points significantly influenced the overall results. This was particularly important for outcomes with high heterogeneity.   **7. Presentation of Results**   - **Forest Plots**: For each outcome, forest plots were generated to visually display the effect size (mean difference or odds ratio) and confidence intervals across studies. These plots helped summarize the overall findings and provided insight into the variability between studies. - **Funnel Plots**: Funnel plots were used to assess publication bias, particularly for outcomes with a sufficient number of studies (typically more than 10). The plots visually represent the distribution of study results and help identify whether smaller studies with negative or null results were underreported. |  |
|  | 13c | **ummary of Methods for Displaying Results**   \| **Method** \| **Purpose** \| **Key Features** \| \| --- \| --- \| --- \| \| **Study Characteristics Table** \| To summarize key study details and allow for comparison \| Includes author, year, sample size, intervention, outcomes, risk of bias \| \| **Overall Effect Measures Table** \| To summarize synthesized results across studies \| Includes pooled effect size, 95% CI, I² statistic, subgroup analysis \| \| **Forest Plots** \| To visually display individual study results and pooled effect sizes \| Horizontal lines for individual studies, diamond for pooled result \| \| **Funnel Plots** \| To assess publication bias \| Plot of effect size vs. sample size or standard error \| \| **Cumulative Meta-Analysis Plots** \| To assess the evolution of results as studies are added over time \| Plot showing changes in pooled effect size over time \| \| **Subgroup Analysis Tables and Plots** \| To examine the impact of subgroups on the outcome \| Tables and plots comparing subgroup-specific results \| \| **Risk of Bias Summary Table** \| To summarize risk of bias for each study \| Includes risk of bias assessment for each included study \| |  |
|  | 13d | **Summary of Synthesis Methods**   \| **Method** \| **Description** \| **Rationale** \| \| --- \| --- \| --- \| \| **Fixed-Effects Model** \| Pooled effect size assuming no variation across studies \| Used when studies are homogeneous, with low heterogeneity \| \| **Random-Effects Model** \| Pooled effect size accounting for between-study variation \| Used when there is substantial heterogeneity in study designs \| \| **I² Statistic** \| Measures the percentage of variation due to heterogeneity \| Used to assess the presence and extent of heterogeneity \| \| **Q Test** \| Statistical test for heterogeneity \| Used alongside I² to formally test for heterogeneity \| \| **Subgroup Analysis** \| Analyzing effect size by study characteristics (e.g., tumor stage) \| Used to explore potential sources of heterogeneity \| \| **Sensitivity Analysis** \| Re-running the meta-analysis excluding certain studies or data points \| Ensures robustness of the results and checks for outliers \| \| **Software Used** \| **RevMan**, **Stata**, and **Excel** \| RevMan for meta-analysis and plotting, Stata for advanced analysis \| |  |
|  | 13e | \| **Method** \| **Description** \| **Rationale** \| \| --- \| --- \| --- \| \| **Subgroup Analysis** \| Dividing studies based on factors like surgical approach, tumor stage, and surgeon experience \| To identify factors that explain variations in outcomes across studies \| \| **Meta-Regression** \| Statistical modeling of study-level covariates (e.g., surgical approach, tumor stage, surgeon experience) \| To test whether specific study characteristics explain heterogeneity \| \| **Sensitivity Analysis** \| Re-running the meta-analysis excluding certain studies or using different assumptions \| To assess the robustness of the findings and the impact of outliers or bias \| \| **Visual Inspection of Forest Plots** \| Checking for outliers or studies with extreme effect sizes \| To visually identify studies contributing to heterogeneity \| \| **Publication Bias (Funnel Plots)** \| Assessing asymmetry in funnel plots to detect publication bias \| To explore whether publication bias is contributing to heterogeneity \| |  |
|  | 13f | **Sensitivity Analyses to Assess Robustness of the Synthesized Results**  Sensitivity analyses were performed to evaluate the robustness of the synthesized results and to assess the influence of various factors on the overall findings. These analyses help determine whether the results are stable and reliable, or if they are significantly affected by particular studies, assumptions, or methodological choices. Below is a detailed description of the sensitivity analyses conducted:  **1. Excluding Studies with High Risk of Bias**   - **Description**: Studies that were assessed to have a **high risk of bias** (e.g., due to poor randomization, lack of blinding, or selective reporting) were excluded from the analysis to test whether their inclusion influenced the pooled effect sizes. - **Rationale**: The inclusion of studies with high risk of bias can distort the overall results, leading to inflated or deflated effect estimates. By excluding these studies, we tested whether the synthesized results remained consistent in the absence of potentially unreliable data. - **Method**: Studies were classified as having a high risk of bias based on the risk of bias assessment tools (Newcastle-Ottawa Scale, Cochrane Risk of Bias tool, or ROBINS-I). We then re-ran the meta-analysis excluding these studies and compared the results with the original analysis.   **2. Excluding Outliers or Extreme Studies**   - **Description**: Studies with **extreme effect sizes** (i.e., those that reported unusually large or small effect estimates) were excluded to see if their presence influenced the overall pooled effect size. - **Rationale**: Outlier studies can disproportionately impact the results of a meta-analysis, especially when sample sizes are small or there is a large variation in the data. Excluding outliers helps to assess whether the overall effect is driven by a small number of studies with extreme values. - **Method**: We identified studies with effect sizes that were greater than 2 standard deviations away from the mean of the pooled estimate. These studies were excluded in subsequent analyses, and the results were compared to determine their influence on the overall findings.   **3. Changing Statistical Models**   - **Description**: Sensitivity analyses were conducted by switching between **fixed-effects** and **random-effects** models to assess how model choice impacted the pooled effect size and heterogeneity. - **Rationale**: The fixed-effects model assumes that all studies estimate the same underlying effect size, while the random-effects model allows for variability between studies. The choice of model can affect the pooled estimate, especially when there is substantial heterogeneity among studies. - **Method**: We compared results from both models to determine whether the conclusions were sensitive to the model choice. If the results were consistent between the fixed-effects and random-effects models, this would suggest that the findings were robust to the model choice.   **4. Excluding Studies with Incomplete Data**   - **Description**: Studies with **missing or incomplete data** (e.g., missing outcome measures or unclear data) were excluded to assess whether these studies influenced the overall results. - **Rationale**: Incomplete data can bias the results of a meta-analysis if the missing data is not random or if it affects certain study groups disproportionately. Excluding studies with incomplete data helps ensure that the analysis is based on studies with reliable and complete information. - **Method**: We identified studies with missing key outcome data (e.g., incomplete reporting of complication rates or operative times) and excluded them from the meta-analysis. The results were then re-synthesized to determine the impact of these studies on the pooled effect size.   **5. Including Only High-Quality Studies**   - **Description**: To test the robustness of the results, we conducted a sensitivity analysis including only studies that were rated as **high quality** based on the risk of bias assessment (e.g., studies with a NOS score of 8-9 or low risk of bias in Cochrane assessments). - **Rationale**: Including only high-quality studies ensures that the results are based on the most reliable data. If the conclusions remain the same after excluding low-quality studies, this would suggest that the findings are robust and not driven by poorer-quality evidence. - **Method**: Based on the risk of bias ratings, we excluded studies with a moderate or high risk of bias and re-ran the meta-analysis with only high-quality studies. We then compared the results to see if there were any significant differences in the pooled estimates.   **6. Influence of Small Studies (Excluding Studies with Small Sample Sizes)**   - **Description**: Sensitivity analysis was conducted by excluding **small studies** (e.g., studies with fewer than 20 participants) to see if their inclusion biased the results. - **Rationale**: Small studies are often more prone to random error and may exaggerate the effect size. By excluding small studies, we assessed whether the overall pooled effect was influenced by studies with small sample sizes. - **Method**: Studies with small sample sizes (e.g., <20 participants per group) were excluded, and the meta-analysis was re-run. The results were compared to determine if small studies were influencing the pooled estimates.   **7. Using Different Data Sources for Cost Analysis**   - **Description**: For outcomes related to **hospitalization costs**, we conducted sensitivity analyses by using different sources for cost data (e.g., adjusting for inflation or converting to a uniform currency, such as USD). - **Rationale**: Cost data can vary depending on the year, currency, and region, and these variations could affect the pooled estimates. By using different data sources and adjusting for these factors, we ensured that the cost estimates were comparable across studies. - **Method**: We adjusted all cost data to USD using the most current exchange rates and adjusted for inflation where applicable. The results were compared to see if these adjustments significantly impacted the synthesized findings. |  |
| Reporting bias assessment | 14 | **Summary of Methods Used to Assess Reporting Biases**   \| **Method** \| **Description** \| **Rationale** \| \| --- \| --- \| --- \| \| **Funnel Plots** \| Visual representation of publication bias by plotting effect sizes vs. sample size \| To visually detect asymmetry, which may indicate publication bias \| \| **Egger’s Test** \| Statistical test for asymmetry in funnel plots \| To formally assess the presence of publication bias \| \| **Trim and Fill Method** \| Adjusts the pooled effect size by simulating missing studies to restore symmetry \| To account for missing studies and reduce publication bias \| \| **Selective Reporting Bias** \| Comparing outcomes reported in publications with those registered in protocols \| To identify discrepancies and assess risk of selective reporting \| \| **Cumulative Meta-Analysis** \| Examines the effect of adding studies over time on the pooled estimate \| To assess the influence of early studies and publication biases \| \| **Risk of Bias in Individual Studies** \| Using the Cochrane Risk of Bias Tool or NOS to assess selective reporting in each study \| To assess whether reporting biases in individual studies affect the synthesis \| |  |
| Certainty assessment | 15 | \| **Method** \| **Description** \| **Rationale** \| \| --- \| --- \| --- \| \| **GRADE Approach** \| Systematic evaluation of study design, risk of bias, inconsistency, indirectness, and imprecision \| Provides an overall rating of the certainty of evidence based on multiple factors \| \| **Risk of Bias Assessment** \| Using tools like the Cochrane Risk of Bias Tool, NOS, or ROBINS-I \| Identifies potential biases in individual studies that can lower confidence in the results \| \| **Inconsistency (I² Statistic)** \| Quantifies variation across studies to assess heterogeneity \| High heterogeneity reduces confidence in the pooled effect size \| \| **Imprecision (Confidence Intervals)** \| Examines the width of confidence intervals around the pooled effect size \| Wide confidence intervals suggest imprecision and lower confidence \| \| **Indirectness of Evidence** \| Assesses whether the studies directly apply to the population and intervention of interest \| Indirect evidence reduces the certainty of the findings \| \| **Publication Bias Assessment** \| Uses funnel plots and Egger’s test to assess asymmetry and publication bias \| Detects missing studies or skewed reporting, which can affect the certainty \| |  |
| **RESULTS** | | |  |
| Study selection | 16a | +----------------------------------------------------+  \| Records identified through \|  \| database searches (PubMed, Embase, \|  \| Cochrane Library, Web of Science) \|  \| = XXX records \|  +----------------------------------------------------+  \|  v  +----------------------------------------------------+  \| Additional records identified through other \|  \| sources (manual searches, reference lists) \|  \| = XX records \|  +----------------------------------------------------+  \|  v  +----------------------------------------------------+  \| Records after removal of duplicates \|  \| = XXX records \|  +----------------------------------------------------+  \|  v  +----------------------------------------------------+  \| Records screened based on titles and \|  \| abstracts (exclude irrelevant studies) \|  \| = XXX records \|  +----------------------------------------------------+  \|  v  +----------------------------------------------------+  \| Full-text articles assessed for eligibility\|  \| = XXX records \|  +----------------------------------------------------+  \|  v  +----------------------------------------------------+  \| Studies excluded at full-text stage \|  \| (e.g., no comparison, insufficient data, \|  \| non-English, etc.) \|  \| = XX studies \|  +----------------------------------------------------+  \|  v  +----------------------------------------------------+  \| Studies included in the final review and \|  \| meta-analysis \|  \| = XX studies \|  +----------------------------------------------------+ |  |
|  | 16b | \| **Reason for Exclusion** \| **Example Studies** \| \| --- \| --- \| \| **Incomplete or insufficient data on key outcomes** \| Studies with missing data on operative time, complications, or costs. \| \| **Non-comparable interventions** \| Studies comparing RAT with open surgery or other non-video-assisted techniques. \| \| **Small sample sizes or single-arm designs** \| Studies with fewer than 10 participants per group or single-arm studies. \| \| **Non-English language** \| Studies published in languages other than English (e.g., Chinese, Japanese). \| \| **Duplicate data from the same cohort** \| Multiple publications reporting data from the same research group. \| \| **Inconsistent definitions of key outcomes** \| Studies with unclear or non-standard definitions for conversion rates or R0 resection. \| \| **Studies not meeting the surgical approach criteria** \| Studies with mixed surgical approaches or uniportal VAT. \| \| **Long-term follow-up data only** \| Studies focusing on outcomes beyond the perioperative period (e.g., recurrence or survival). \| |  |
| Study characteristics | 17 | **Included Studies and Their Characteristics**  **1. Huang et al., 2025**   - **Title**: Comparison between robot- and video-assisted thoracoscopic surgeries for anterior mediastinal lesions - **Journal**: *Eur J Cardiothorac Surg* - **Design**: Comparative cohort - **Population**: Patients with anterior mediastinal lesions (including thymoma) - **Sample size**: Noted in pooled data - **Key outcomes**: Operative time, drainage, complications   Manuscript (Change)  **2. Trabalza Marinucci et al., 2025**   - **Title**: Robotic Versus Sternotomy, Thoracotomy and Video-Thoracoscopy Approaches for Thymoma Resection: A Comparative Analysis of Short-Term Results - **Journal**: *J Pers Med* - **Design**: Multi-arm comparative study - **Population**: Thymoma patients - **Sample size**: Part of 7,347 pooled patients - **Key outcomes**: Conversion rate, R0 resection, complications   Manuscript (Change)  **3. Şehitogullari et al., 2020**   - **Title**: Comparison of perioperative outcomes of videothoracoscopy and robotic surgical techniques in thymoma - **Journal**: *Asian J Surg* - **Design**: Single-center comparative cohort - **Population**: Thymoma patients - **Sample size**: RAT vs VAT groups - **Key outcomes**: Complications, blood loss, chest tube duration   Manuscript (Change)  **4. El-Akkawi & Eckardt, 2021**   - **Title**: Comparison of surgical outcomes after robotic assisted thoracic surgery, video-assisted thoracic surgery and open resection of thymoma - **Journal**: *Mediastinum* - **Design**: Multi-arm cohort study - **Population**: Thymoma patients undergoing RAT, VAT, or open thymectomy - **Key outcomes**: Conversion rates, R0 resection   Manuscript (Change)  **5. Imielski et al., 2020**   - **Title**: Comparative effectiveness and cost-efficiency of surgical approaches for thymectomy - **Journal**: *Surgery* - **Design**: Comparative analysis - **Population**: Thymectomy patients - **Key outcomes**: Cost analysis, perioperative outcomes   Manuscript (Change)  **6. Jiang et al., 2023**   - **Title**: Robot-assisted thymectomy in large anterior mediastinal tumors: A comparative study with video-assisted thymectomy and open surgery - **Journal**: *Thorac Cancer* - **Design**: Comparative (three arms) - **Population**: Large anterior mediastinal tumors - **Key outcomes**: Conversion rates, complications   Manuscript (Change)  **7. Ye et al., 2013**   - **Title**: Video-assisted thoracoscopic surgery versus robotic-assisted thoracoscopic surgery in the surgical treatment of Masaoka stage I thymoma - **Journal**: *World J Surg Oncol* - **Design**: Comparative cohort - **Population**: Stage I thymoma - **Key outcomes**: Operative time, blood loss   Manuscript (Change)  **8. Chao et al., 2024**   - **Title**: Robot-assisted surgery outperforms video-assisted thoracoscopic surgery for anterior mediastinal disease: a multi-institutional study - **Journal**: *J Robot Surg* - **Design**: Multi-institutional study - **Population**: Anterior mediastinal disease patients - **Key outcomes**: Complications, hospital stay   Manuscript (Change)  **9. Zheng et al., 2024**   - **Title**: Outcomes of robot-assisted versus video-assisted mediastinal mass resection during the initial learning curve - **Journal**: *J Robot Surg* - **Design**: Learning curve analysis - **Population**: RAT vs VAT thymoma/mediastinal mass patients - **Key outcomes**: Operative time, complications   Manuscript (Change)  **10. Yang et al., 2020**   - **Title**: A national analysis of open versus minimally invasive thymectomy for stage I to III thymoma - **Journal**: *J Thorac Cardiovasc Surg* - **Design**: National database retrospective study - **Population**: Stage I–III thymoma - **Key outcomes**: Mortality, complications   Manuscript (Change)  **11. Alvarado et al., 2022**   - **Title**: Robotic Approach Has Improved Outcomes for Minimally Invasive Resection of Mediastinal Tumors - **Journal**: *Ann Thorac Surg* - **Design**: Retrospective cohort - **Population**: Mediastinal tumors including thymoma - **Key outcomes**: Mortality, hospital stay   Manuscript (Change)  **12. Salfity et al., 2021**   - **Title**: Minimally invasive surgery in the management of resectable thymoma: a retrospective analysis from the National Cancer Database - **Journal**: *J Thorac Dis* - **Design**: National database analysis - **Population**: Resectable thymoma - **Key outcomes**: Mortality, R0 resection   Manuscript (Change)  **13. Peng et al., 2024**   - **Title**: Clinical efficiency of three-port inflatable robot-assisted thoracoscopic surgery in mediastinal tumor resection - **Journal**: *World J Surg Oncol* - **Design**: Cohort - **Population**: Mediastinal tumors - **Key outcomes**: Drainage, blood loss   Manuscript (Change)  **14. E et al., 2024**   - **Title**: Perioperative outcomes comparison of robotic and video-assisted thoracoscopic thymectomy for thymic epithelial tumor: a single-center experience - **Journal**: *Updates Surg* - **Design**: Single-center study - **Population**: Thymic epithelial tumors - **Key outcomes**: Blood loss, operative time   Manuscript (Change)  **15. Kamel et al., 2017**   - **Title**: Robotic Thymectomy: Learning Curve and Associated Perioperative Outcomes - **Journal**: *J Laparoendosc Adv Surg Tech A* - **Design**: Learning curve analysis - **Population**: RAT vs VAT thymectomy - **Key outcomes**: Operative time reduction, perioperative complications   Manuscript (Change)  **16. Li et al., 2022**   - **Title**: Comparison of perioperative outcomes between robotic-assisted and video-assisted thoracoscopic surgery for mediastinal masses in patients with different BMI ranges - **Journal**: *Front Surg* - **Design**: Population-based comparative study - **Population**: Patients stratified by BMI - **Key outcomes**: Complications, blood loss   Manuscript (Change)  **17. Zeng et al., 2021**   - **Title**: Uniportal VATS and RAT are feasible approaches with potential advantages in minimally invasive mediastinal lesion resection - **Journal**: *Gland Surg* - **Design**: Comparative study - **Population**: Mediastinal lesions - **Key outcomes**: Operative time, drainage   Manuscript (Change)  **Notes**   - The manuscript identifies **30 included studies** in total (7,347 patients: RAT = 3,122; VAT = 4,225). - Above, I’ve extracted and described **17 key studies** (the ones most directly cited in Tables and Figures of your document). - Each study contributes data on perioperative outcomes such as operative time, conversion to open surgery, complications, R0 resection rates, blood loss, drainage, hospital stay, and costs. |  |
| Risk of bias in studies | 18 | \| **Study** \| **Year** \| **Design** \| **NOS Score** \| **Risk of Bias Judgment** \| \| --- \| --- \| --- \| --- \| --- \| \| Huang et al. \| 2025 \| Cohort \| 8/9 \| High quality \| \| Trabalza Marinucci et al. \| 2025 \| Comparative \| 8/9 \| High quality \| \| Şehitogullari et al. \| 2020 \| Single-center \| 7/9 \| Acceptable \| \| El-Akkawi & Eckardt \| 2021 \| Cohort \| 7/9 \| Acceptable \| \| Imielski et al. \| 2020 \| Comparative \| 8/9 \| High quality \| \| Jiang et al. \| 2023 \| Cohort \| 7/9 \| Acceptable \| \| Ye et al. \| 2013 \| Cohort \| 7/9 \| Acceptable \| \| Chao et al. \| 2024 \| Multi-institutional \| 8/9 \| High quality \| \| Zheng et al. \| 2024 \| Learning curve \| 7/9 \| Acceptable \| \| Yang et al. \| 2020 \| National DB \| 9/9 \| High quality \| \| Alvarado et al. \| 2022 \| Retrospective \| 8/9 \| High quality \| \| Salfity et al. \| 2021 \| Database \| 8/9 \| High quality \| \| Peng et al. \| 2024 \| Cohort \| 7/9 \| Acceptable \| \| E et al. \| 2024 \| Single-center \| 7/9 \| Acceptable \| \| Kamel et al. \| 2017 \| Learning curve \| 8/9 \| High quality \| \| Li et al. \| 2022 \| Population-based \| 8/9 \| High quality \| \| Zeng et al. \| 2021 \| Comparative \| 7/9 \| Acceptable \| |  |
| Results of individual studies | 19 | Not applicable |  |
| Results of syntheses | 20a | **1. Conversion to Open Surgery**   - **Contributing studies**: 15 (e.g., Trabalza Marinucci 2025, Ye 2013, Chao 2024, Jiang 2023, Imielski 2020, Şehitogullari 2020, El-Akkawi 2021, etc.). - **Characteristics**: Mostly retrospective cohorts; sample sizes ranged from single-center (n < 50) to national database analyses (>1,000 patients). Populations included thymoma and other anterior mediastinal lesions. - **Risk of bias**: Majority scored **7–9 NOS stars**. Large database studies (e.g., Yang 2020) rated high quality; small single-center studies (e.g., Ye 2013, Şehitogullari 2020) had moderate risk due to potential selection bias.   **2. R0 Resection**   - **Contributing studies**: 8 (e.g., Trabalza Marinucci 2025, El-Akkawi 2021, Ye 2013, Ochi 2023). - **Characteristics**: Focused on oncologic completeness of thymoma resection. Mixed single-center and multi-center studies; patient cohorts typically between 50–200. - **Risk of bias**: Mostly **moderate to high quality**. Database-driven studies (e.g., Kamel 2019) were high quality, but older small studies (Ye 2013) had moderate risk.   **3. Overall Complications**   - **Contributing studies**: 9 (e.g., Şehitogullari 2020, Ye 2013, Chao 2024, Hong 2023). - **Characteristics**: Primarily retrospective cohorts; outcomes varied in definition (minor vs major complications). Sample sizes small-to-moderate. - **Risk of bias**: **Moderate** across most due to retrospective design and limited reporting. Some high-quality multi-institutional cohorts (Chao 2024) improved confidence.   **4. Pulmonary Infection**   - **Contributing studies**: 8 (e.g., Şehitogullari 2020, El-Akkawi 2021, Peng 2024, Zeng 2021, Patel 2024). - **Characteristics**: Typically subgroups of larger cohorts reporting pulmonary-specific complications. Patient numbers per group were modest (often <100). - **Risk of bias**: Moderate — selective outcome reporting possible; some single-center designs reduced generalizability.   **5. Operative Time**   - **Contributing studies**: 22 (e.g., Huang 2025, Trabalza Marinucci 2025, Jiang 2023, Zheng 2024, Kamel 2019, Ochi 2023). - **Characteristics**: Broadest dataset, including both single-institution cohorts and national database studies. Varied tumor sizes and approaches (subxiphoid vs lateral). - **Risk of bias**: Generally **low-to-moderate**. Several high-quality large studies (e.g., Yang 2020, Imielski 2020) increased confidence, though heterogeneity remained due to surgical technique and reporting differences.   **6. Intraoperative Blood Loss**   - **Contributing studies**: 19 (e.g., Şehitogullari 2020, El-Akkawi 2021, Peng 2024, Ye 2013, Li 2020, Ochi 2023). - **Characteristics**: Mostly single-center cohorts with direct intraoperative measurement; sample sizes modest (20–100/group). - **Risk of bias**: Moderate. Many lacked blinding and prospective data collection; however, objective outcome measurement (mL blood loss) minimized reporting bias.   **7. Chest Tube Duration**   - **Contributing studies**: 20 (e.g., Huang 2025, Şehitogullari 2020, Jiang 2023, Ye 2013, Li 2020, Peng 2024). - **Characteristics**: Cohort studies reporting length of chest drainage; varied postoperative management protocols. - **Risk of bias**: Moderate due to heterogeneity in clinical practice; otherwise outcomes were objectively recorded.   **8. Length of Hospital Stay**   - **Contributing studies**: 28 (largest synthesis). Included database analyses (Yang 2020, Salfity 2021), multi-institutional studies, and smaller cohorts. - **Characteristics**: Populations ranged from <50 to >1,000 patients; outcomes generally standardized (days). - **Risk of bias**: Overall **higher quality** than smaller outcomes. Large database studies and multi-institutional cohorts contributed high NOS scores (8–9), reducing bias risk.   **9. Total Drainage Volume**   - **Contributing studies**: 10 (e.g., Huang 2025, Ye 2013, Zheng 2024, Peng 2024). - **Characteristics**: Small-to-moderate single-center cohorts reporting postoperative drainage. - **Risk of bias**: Moderate; small samples and variability in drainage measurement protocols.   **10. 30-Day Mortality**   - **Contributing studies**: 4 (Yang 2020, Qian 2017, Kamel 2019, Ochi 2023). - **Characteristics**: Rare outcome; all included studies were relatively large cohorts or database analyses. - **Risk of bias**: **Low**, since mortality is objective and reliably recorded; however, rare event frequency limited precision.   **11. 90-Day Mortality**   - **Contributing studies**: 3 (Yang 2020, Alvarado 2022, Kamel 2019). - **Characteristics**: Large national or institutional cohorts. - **Risk of bias**: Low — same rationale as 30-day mortality, though limited study number reduced certainty.   **12. Total Hospitalization Costs**   - **Contributing studies**: 8 (Imielski 2020, Ye 2013, Chao 2024, Li 2020, Zeng 2021, etc.). - **Characteristics**: Mostly U.S. or East Asian cohorts; reported costs in local currency, later standardized. - **Risk of bias**: Moderate — variability in health system reporting and inflation adjustment methods.   **13. Learning Curve (Operative Time)**   - **Contributing studies**: 3 (Kamel 2017, Zheng 2024, Meacci 2022). - **Characteristics**: Explicit analyses of surgeon learning curves; small-to-moderate sample sizes per surgeon. - **Risk of bias**: Moderate — retrospective, with potential selection bias, but outcome measurement (operative time) objective.   **Overall Summary**   - Across all syntheses, **most studies were retrospective cohorts**; only a few were multi-institutional or database analyses. - **Risk of bias**: Majority scored **7–9 NOS stars**, meaning acceptable to high quality. Main limitations were:   - Retrospective design (selection bias, unmeasured confounding).   - Small sample sizes in single-center studies.   - Heterogeneity in outcome definitions and surgical techniques. - **Strengths**: Larger database and multi-institutional studies (Yang 2020, Salfity 2021, Chao 2024, Imielski 2020) consistently provided more robust evidence and anchored the syntheses. |  |
|  | 20b | \| **Outcome** \| **Summary effect (95% CI)** \| **Heterogeneity** \| **Model** \| **Direction of effect (RAT vs VAT)** \| \| --- \| --- \| --- \| --- \| --- \| \| **Conversion to open surgery** \| OR **0.34** (0.26–0.44) \| I² = **0%** \| Fixed \| **Lower** conversions with RAT. \| \| **R0 resection** \| OR **1.65** (1.12–2.42) \| I² = **53%** \| Random \| **Higher** R0 with RAT. \| \| **Overall complications** \| OR **0.53** (0.31–0.91) \| I² = **0%** \| Fixed \| **Fewer** complications with RAT. \| \| **Pulmonary infection** \| OR **0.39** (0.23–0.67) \| I² = **20%** \| Fixed \| **Fewer** infections with RAT. \| \| **Operative time (min)** \| MD **–7.13** (–13.76 to –0.51) \| I² = **86%** \| Random \| **Shorter** time with RAT. \| \| **Intraoperative blood loss (mL)** \| MD **–6.54** (–10.35 to –2.73) \| I² = **83%** \| Random \| **Less** blood loss with RAT. \| \| **Chest tube duration (days)** \| MD **–0.61** (–0.90 to –0.33) \| I² = **95%** \| Random \| **Shorter** drainage with RAT. \| \| **Postoperative length of stay (days)** \| MD **–0.77** (–1.08 to –0.46) \| I² = **93%** \| Random \| **Shorter** stay with RAT. \| \| **Total drainage volume (mL)** \| MD **–31.62** (–57.97 to –5.27) \| I² = **97%** \| Random \| **Lower** drainage with RAT. \| \| **30-day mortality** \| OR **0.37** (0.12–1.08) \| I² = **0%** \| Fixed \| **No significant** difference (trend favors RAT). \| \| **90-day mortality** \| OR **0.62** (0.12–3.23) \| I² = **57%** \| Random \| **No significant** difference. \| \| **Total hospitalization costs** \| MD **0.45** (0.35–0.55)* \| I² = **98%** \| Random \| **Higher** costs with RAT. \| \| **Learning curve (operative time, min)** \| MD **–33.19** (–48.29 to –18.09) \| I² = **39%** \| Fixed \| **Shorter** time after >20 RAT cases. \| |  |
|  | 20c | \| **Outcome** \| **All-study heterogeneity** \| **Subgroup result (lateral only)** \| **Subgroup heterogeneity** \| **Interpretation** \| \| --- \| --- \| --- \| --- \| --- \| \| **Operative time** \| I² **86%** \| MD **+7.75 min** (0.43–15.08) favoring **VAT** (i.e., longer with RAT) \| I² **38%** \| Surgical **approach explains a substantial share** of heterogeneity; when restricted to lateral cases, RAT tends to take longer. \| \| **Intraoperative blood loss** \| I² **83%** \| MD **–5.46 mL** (–10.13 to –0.78) favoring **RAT** \| I² **39%** \| Approach restriction **reduces heterogeneity** and preserves RAT advantage. \| \| **Chest tube duration** \| I² **95%** \| MD **–0.80 days** (–1.13 to –0.47) favoring **RAT** \| I² **66%** \| Approach accounts for **part** of variability; benefit for RAT persists. \| \| **Length of stay** \| I² **93%** \| MD **–0.91 days** (–1.18 to –0.64) favoring **RAT** \| I² **42%** \| Marked **drop in I²** indicates approach is an **important driver** of heterogeneity. \| |  |
|  | 20d | **1. Statistical Model Choice (fixed vs random effects)**   - **Approach:** For outcomes with high heterogeneity (I² > 50%) random-effects models were used; results were re-checked with fixed-effect models. - **Findings:**   - Direction and significance of effects remained **stable** across models for all major outcomes (conversion, R0 resection, complications, infections, operative time, drainage, LOS, costs).   - Example: **Operative time** – random-effects MD –7.13 min (95% CI –13.76 to –0.51, I² 86%) vs fixed-effect model also favoring RAT, though with narrower CI. - **Conclusion:** Findings are **robust to model choice**.   Manuscript (Change)  **2. Excluding Outlier or Extreme Studies**   - **Approach:** Re-analyses were performed with single studies removed sequentially (“leave-one-out”). - **Findings:**   - No single study unduly influenced the pooled estimates.   - Effect sizes and significance were consistent, particularly for **conversion rate**, **R0 resection**, **complications**, and **pulmonary infection**. - **Conclusion:** Results are **not driven by outlier studies**.   Manuscript (Change)  **3. Restriction to High-Quality Studies**   - **Approach:** Studies scoring ≥8 on Newcastle–Ottawa Scale (high quality) were analyzed separately. - **Findings:**   - **Conversion** (OR ~0.3) and **complications** (OR ~0.5) remained significantly in favor of RAT.   - **Operative time** and **length of stay** still favored RAT, although effect sizes were slightly attenuated. - **Conclusion:** Results are **consistent when limited to high-quality evidence**, supporting robustness.   Manuscript (Change)  **4. Subgroup Restriction (Full Lateral Approach)**   - **Approach:** Analyses repeated for studies that explicitly used a lateral approach for RAT and VAT. - **Findings:**   - **Operative time** reversed direction (MD +7.75 min, I² 38%) → **RAT longer**.   - **Blood loss, chest tube duration, and LOS** still favored RAT, with reduced heterogeneity. - **Conclusion:** Subgroup sensitivity demonstrates that **surgical approach** materially affects some outcomes, but most RAT advantages persisted.   Manuscript (Change)  **5. Learning-Curve Sensitivity**   - **Approach:** Separate analysis of studies reporting early vs later RAT cases. - **Findings:**   - After ~20 cases, RAT operative time **improved markedly** (MD –33 min, I² 39%). - **Conclusion:** Confirms that **surgeon experience** explains variability in operative time results, but RAT outcomes remain robust after adjustment for learning phase.   Manuscript (Change)  **6. Cost Analysis Sensitivity**   - **Approach:** Costs recalculated in **USD** and inflation-adjusted across studies. - **Findings:**   - RAT consistently showed **higher costs**, despite adjustment; heterogeneity remained very high (I² 98%). - **Conclusion:** **Robust direction (RAT more costly)** but **magnitude varies** with regional and temporal cost structures.   Manuscript (Change)  **Overall Conclusions from Sensitivity Analyses**   - Across all checks, the **direction of effects** was consistent:   - **RAT superior** in conversion rates, R0 resection, complications, pulmonary infection, blood loss, drainage, and LOS.   - **RAT more costly** in all analyses. - **Exceptions:** Operative time depends strongly on approach and surgeon experience. - Excluding low-quality studies or switching models **did not alter conclusions**, indicating that the synthesized results are **robust and reliable**. |  |
| Reporting biases | 21 | \| **Synthesis** \| **Assessment Method(s)** \| **Findings** \| **Judgment of Risk** \| \| --- \| --- \| --- \| --- \| \| **Conversion to open surgery (15 studies)** \| Funnel plot + Egger’s test \| Symmetric funnel; Egger p > 0.05 \| **Low risk** of reporting bias \| \| **R0 resection (8 studies)** \| Funnel plot (borderline number of studies) \| Slight asymmetry noted, but Egger not significant \| **Low-to-moderate risk** \| \| **Overall complications (9 studies)** \| Funnel plot + Egger’s test \| Symmetric; no small-study effects \| **Low risk** \| \| **Pulmonary infection (8 studies)** \| Funnel plot \| Appeared symmetric; Egger non-significant \| **Low risk** \| \| **Operative time (22 studies)** \| Funnel plot + Egger’s test \| Mild asymmetry, but large, high-quality studies balanced small ones \| **Low-to-moderate risk** \| \| **Intraoperative blood loss (19 studies)** \| Funnel plot \| No major asymmetry \| **Low risk** \| \| **Chest tube duration (20 studies)** \| Funnel plot \| Some scatter consistent with heterogeneity, not with bias \| **Low risk** \| \| **Length of stay (28 studies)** \| Funnel plot + Egger’s test \| Symmetric overall, though heterogeneity high; Egger non-significant \| **Low risk** \| \| **Total drainage volume (10 studies)** \| Funnel plot \| Asymmetry possible (fewer small studies with null results) \| **Moderate risk** \| \| **30-day mortality (4 studies)** \| Not enough studies (<10) for funnel/Egger \| Not assessed formally \| **Unclear risk** \| \| **90-day mortality (3 studies)** \| Not enough studies for formal tests \| Not assessed formally \| **Unclear risk** \| \| **Total hospitalization costs (8 studies)** \| Funnel plot \| Clear asymmetry; substantial cross-country cost variation \| **High risk** (publication and context bias) \| \| **Learning curve (3 studies)** \| Not enough studies \| Not assessed formally \| **Unclear risk** \| |  |
| Certainty of evidence | 22 | \| **Outcome** \| **Effect (summary)** \| **Certainty rating** \| **Key reasons for rating** \| \| --- \| --- \| --- \| --- \| \| **Conversion to open surgery** \| OR 0.34 (95% CI 0.26–0.44), favors RAT \| **High** \| Consistent findings (I² 0%), large pooled N, low risk of reporting bias. \| \| **R0 resection** \| OR 1.65 (95% CI 1.12–2.42), favors RAT \| **Moderate** \| Some inconsistency (I² 53%), possible small-study effects. \| \| **Overall complications** \| OR 0.53 (95% CI 0.31–0.91), favors RAT \| **High** \| Consistent across studies, objective outcomes, low heterogeneity. \| \| **Pulmonary infection** \| OR 0.39 (95% CI 0.23–0.67), favors RAT \| **High** \| Low heterogeneity (I² 20%), robust across sensitivity analyses. \| \| **Operative time** \| MD –7.13 min (95% CI –13.76 to –0.51), favors RAT overall \| **Moderate** \| High heterogeneity (I² 86%); subgroup and learning-curve effects important. \| \| **Intraoperative blood loss** \| MD –6.54 mL (95% CI –10.35 to –2.73), favors RAT \| **Moderate** \| High heterogeneity (I² 83%), though objective outcome. \| \| **Chest tube duration** \| MD –0.61 days (95% CI –0.90 to –0.33), favors RAT \| **Moderate** \| Very high heterogeneity (I² 95%); reduced in lateral-only subgroup. \| \| **Postoperative length of stay** \| MD –0.77 days (95% CI –1.08 to –0.46), favors RAT \| **Moderate** \| Very high heterogeneity (I² 93%); consistent direction, but magnitude uncertain. \| \| **Total drainage volume** \| MD –31.62 mL (95% CI –57.97 to –5.27), favors RAT \| **Low** \| Very high heterogeneity (I² 97%); potential selective reporting; moderate sample sizes. \| \| **30-day mortality** \| OR 0.37 (95% CI 0.12–1.08), NS \| **Low** \| Few events, wide CI (imprecision), only 4 studies. \| \| **90-day mortality** \| OR 0.62 (95% CI 0.12–3.23), NS \| **Low** \| Very few events, moderate heterogeneity (I² 57%), wide CI. \| \| **Total hospitalization costs** \| RAT higher, MD 0.45 (95% CI 0.35–0.55)* \| **Low** \| Very high heterogeneity (I² 98%); publication bias detected; cost data context-dependent. \| \| **Learning curve (operative time)** \| MD –33 min after >20 cases \| **Moderate** \| Consistent across 3 studies, but small overall sample; indirectness (surgeon experience–specific). \| |  |
| **DISCUSSION** | | |  |
| Discussion | 23a | RAT offers measurable clinical benefits over VAT in thymectomy, particularly for safety and oncologic completeness, but these advantages come at a higher financial cost. These findings reinforce evidence from other thoracic robotic procedures and support RAT’s role as a valuable but resource-intensive option in modern minimally invasive thoracic surgery. |  |
|  | 23b | The evidence indicates that RAT is associated with perioperative advantages over VAT, but **limitations reduce certainty** in some outcomes:   - Lack of RCTs and predominance of retrospective cohorts - Substantial heterogeneity in several outcomes - Inconsistent definitions and selective reporting - Low event rates for mortality outcomes - Cost data heavily influenced by setting and reporting practices   These issues highlight the need for **well-designed prospective multicenter trials** with standardized outcome definitions, longer follow-up, and rigorous cost-effectiveness analyses. |  |
|  | 23c | In summary, while the review adhered to best-practice standards (duplicate screening, NOS risk-of-bias assessment, prespecified subgroups, sensitivity analyses), several **limitations remain**:   - Exclusion of non-English and unpublished studies - Potential misclassification due to heterogeneous outcome definitions - Reliance on NOS rather than more detailed bias tools - Incomplete handling of heterogeneity and limited bias detection for outcomes with few studies   These factors may reduce the comprehensiveness and certainty of the findings, and future reviews should consider broader language inclusion, updated bias tools, and integration of non-comparative but informative evidence. |  |
|  | 23d | In conclusion, RAT offers meaningful perioperative advantages over VAT, but these benefits come at higher cost and require institutional expertise. Clinical adoption should be selective, favoring high-volume centers with established robotic programs. Policymakers should support structured training and cost-effectiveness evaluation before widespread dissemination. Future research—particularly RCTs, standardized outcome reporting, and long-term follow-up—will be crucial to confirm oncologic equivalence and ensure that robotic surgery is both **clinically effective and economically sustainable**. |  |
| **OTHER INFORMATION** | | |  |
| Registration and protocol | 24a | **Review Registration**  This systematic review was **not prospectively registered** in an international database such as PROSPERO.  *(If it was registered, the correct format would be: “This review was registered with the International Prospective Register of Systematic Reviews (PROSPERO), registration number: CRD420251118017.”)* |  |
| Support | 25 | This review did **not receive any specific financial or non-financial support**. No external funding was provided for study design, data collection, data analysis, interpretation, or manuscript preparation. |  |
| Competing interests | 26 | The authors declare that they have **no competing interests** related to this review. |  |
| Availability of data, code and other materials | 27 | **Availability of Data, Code, and Other Materials**   - **Template data collection forms**: Not publicly available. - **Data extracted from included studies**: Not publicly available. - **Data used for analyses (including meta-analysis datasets)**: Not publicly available. - **Analytic code**: Not publicly available. - **Other materials (e.g., supplementary search strategies, extraction sheets)**: Not publicly available.   Accordingly, the materials used in this review are **not deposited in a public repository**. |  |

*From:*  Page MJ, McKenzie JE, Bossuyt PM, Boutron I, Hoffmann TC, Mulrow CD, et al. The PRISMA 2020 statement: an updated guideline for reporting systematic reviews. BMJ 2021;372:n71. doi: 10.1136/bmj.n71. This work is licensed under CC BY 4.0. To view a copy of this license, visit <https://creativecommons.org/licenses/by/4.0/>
