# Supplementary material for: Comparative meta-analysis of robot- and video-assisted surgery for thymoma: efficacy, learning curve, and economic burden in 7347 patients
Source: World J Surg Oncol. 2025 Nov 29;24:14. doi: 10.1186/s12957-025-04132-2 (PMC12771877; doi:10.1186/s12957-025-04132-2)
Supplement: Supplementary file 2 — Supplementary Material 2 [file 12957_2025_4132_MOESM2_ESM.pdf]

NIHR | National Institute for Health and Care Research

PROSPERO
International prospective register of systematic reviews

Robot-assisted Thoracic Surgery versus Video-assisted Thoracic Surgery in Thymoma Treatment: A Meta-analysis of Clinical Efficacy, Learning Curve, and Medical Costs Based on 7347 Patients

Guohang Shen, Haobo Zhang

Citation
Guohang Shen, Haobo Zhang. Robot-assisted Thoracic Surgery versus Video-assisted Thoracic Surgery in Thymoma Treatment: A Meta-analysis of Clinical Efficacy, Learning Curve, and Medical Costs Based on 7347 Patients. PROSPERO 2025 CRD420251118017. Available from https://www.crd.york.ac.uk/PROSPERO/view/CRD420251118017.

REVIEW TITLE AND BASIC DETAILS

Review title
Robot-assisted Thoracic Surgery versus Video-assisted Thoracic Surgery in Thymoma Treatment: A Meta-analysis of Clinical Efficacy, Learning Curve, and Medical Costs Based on 7347 Patients

Condition or domain being studied
thymoma

Rationale for the review
Find the differences between robotic surgery and thoracoscopic surgery in clinical treatment

Review objectives
30 - day and 90 - day mortality rates，The treatment effect during the perioperative period

Keywords
Thymoma; Robotic surgery; Video-assisted thoracoscopic surgery

Country
China

ELIGIBILITY CRITERIA

Population

Included
Include all patients with thymoma

Excluded
Exclude patients under 18 years old and over 70 years old.

Intervention(s) or exposure(s)

Included
Thyme; Robotic assisted surgery; Video Assisted Thoracoscopy

Comparator(s) or control(s)

Included
PICO tags selected: Robotic assisted surgery; Robotic Surgical System; Robot

Study design
Both randomized and nonrandomized study types will be included.

Context
Only include patients with thymoma.

TIMELINE OF THE REVIEW

Date of first submission to PROSPERO
02 August 2025

Review timeline
Start date: 6 June 2025. End date: 2 October 2025.

Date of registration in PROSPERO
02 August 2025

AVAILABILITY OF FULL PROTOCOL

Availability of full protocol
A full protocol has not been written.

SEARCHING AND SCREENING

Search for unpublished studies
Only published studies will be sought.

Main bibliographic databases that will be searched
The main databases to be searched are *CLIB - The Cochrane Library*, *Embase - Embase via Ovid*, *Embase.com*, *MEDLINE*, *PubMed*, *SCI - Science Citation Index* and *Scopus*.

Search language restrictions

The review will only include studies published in English.

Search date restrictions

Databases will be searched for articles published from 2 August 2013 and before by 2 August 2025.

Other methods of identifying studies

Other studies will be identified by: *contacting authors or experts*, *reference list checking* and *searching dissertation and thesis databases*.

Link to search strategy

A full search strategy has been uploaded to PROSPERO. The PDF may be accessed through this link https://www.crd.york.ac.uk/PROSPEROFILES/74762527fa42b6881fea61dbfe2cf27c.pdf.

Selection process

Studies will be screened independently by at least two people (or person/machine combination) with a process to resolve differences.

Other relevant information about searching and screening

None

DATA COLLECTION PROCESS

Data extraction from published articles and reports

Data will be extracted independently by at least two people (or person/machine combination) with a process to resolve differences.

Authors will not be contacted for further information.

Study risk of bias or quality assessment

Risk of bias will be assessed using: *Cochrane RoB-2* and *Newcastle-Ottawa*

Data will be assessed independently by at least two people (or person/machine combination) with a process to resolve differences.

Additional information will be sought from study investigators if required information is unclear or unavailable in the study publications/reports.

Reporting bias assessment

Risk of bias due to missing results will be assessed

Certainty assessment

Certainty of findings will not be assessed

OUTCOMES TO BE ANALYSED

Main outcomes

Ro resection rate,30-day mortality rate

Additional outcomes

The cost of hospitalization

PLANNED DATA SYNTHESIS

Strategy for data synthesis

No formal data synthesis is planned - data will be described but not combined.

CURRENT REVIEW STAGE

Stage of the review at this submission

Review stage	Started	Completed
Pilot work		
Formal searching/study identification		
Screening search results against inclusion criteria		
Data extraction or receipt of IPD		
Risk of bias/quality assessment		
Data synthesis		

Review status

The review is currently planned or ongoing.

Publication of review results

Results of the review will be published.

REVIEW AFFILIATION, FUNDING AND PEER REVIEW

Review team members

Mr Guohang Shen (review guarantor and contact) Ningxia Medical University. China.

No conflict of interest declared.

Dr Haobo Zhang. Ningxia Medical University. China.

No conflict of interest declared.

Named contact

Mr Guohang Shen (mailtosgh@163.com). Ningxia Medical University. China.

Review affiliation

Ningxia Medical University

Funding source

Review has no specific/external funding but is supported by guarantor/review team (non-commercial) institutions.

Peer review

There has been no peer review of this planned review.

ADDITIONAL INFORMATION

Review conflict of interest
Declared individual interests are recorded under team member details.. No additional interests are recorded for this review.

Medical Subject Headings
Humans

SIMILAR REVIEWS

Check for similar records already in PROSPERO
PROSPERO identified a number of existing PROSPERO records that were similar to this one (last check made on 2 August 2025). These are shown below along with the reasons given by that the review team for the reviews being different and/or proceeding.

- Robotic-assisted surgery compared with laparoscopic / video assisted thoracoscopic and open approaches for cancer indications: A systematic review and meta-analysis [published 6 October 2023] [CRD42023466759]. The review was judged **not to be similar**
- Comparison of robot-assisted thoracic surgery versus video-assisted thoracic surgery in the treatment of lung cancer: A systematic review and meta-analysis of prospective Studies [published 29 July 2023] [CRD42023446653]. The review was judged **not to be similar**
- Comparison of robot-assisted thoracic surgery versus video-assisted thoracic surgery in the treatment of lung cancer: A systematic review and meta-analysis of randomized controlled trials [published 29 July 2023] [CRD42023446392]. The review was judged **not to be similar**

PROSPERO version history

- Version 1.0, published 02 Aug 2025

Disclaimer
The content of this record displays the information provided by the review team. PROSPERO does not peer review registration records or endorse their content.

PROSPERO accepts and posts the information provided in good faith; responsibility for record content rests with the review team. The guarantor for this record has affirmed that the information provided is truthful and that they understand that deliberate provision of inaccurate information may be construed as scientific misconduct.

PROSPERO does not accept any liability for the content provided in this record or for its use. Readers use the information provided in this record at their own risk.

Any enquiries about the record should be referred to the named review contact
